# Therapeutic Targeting of Notch Signaling: From Cancer to Inflammatory Disorders

**DOI:** 10.3389/fcell.2021.649205

**Published:** 2021-05-28

**Authors:** Frederick Allen, Ivan Maillard

**Affiliations:** ^1^Division of Hematology and Oncology, Department of Medicine, Perelman School of Medicine at the University of Pennsylvania, Philadelphia, PA, United States; ^2^Abramson Family Cancer Research Institute, Perelman School of Medicine at the University of Pennsylvania, Philadelphia, PA, United States

**Keywords:** Notch, Notch ligands, immune system, inflammation, cancer

## Abstract

Over the past two decades, the Notch signaling pathway has been investigated as a therapeutic target for the treatment of cancers, and more recently in the context of immune and inflammatory disorders. Notch is an evolutionary conserved pathway found in all metazoans that is critical for proper embryonic development and for the postnatal maintenance of selected tissues. Through cell-to-cell contacts, Notch orchestrates cell fate decisions and differentiation in non-hematopoietic and hematopoietic cell types, regulates immune cell development, and is integral to shaping the amplitude as well as the quality of different types of immune responses. Depriving some cancer types of Notch signals has been shown in preclinical studies to stunt tumor growth, consistent with an oncogenic function of Notch signaling. In addition, therapeutically antagonizing Notch signals showed preclinical potential to prevent or reverse inflammatory disorders, including autoimmune diseases, allergic inflammation and immune complications of life-saving procedures such allogeneic bone marrow and solid organ transplantation (graft-versus-host disease and graft rejection). In this review, we discuss some of these unique approaches, along with the successes and challenges encountered so far to target Notch signaling in preclinical and early clinical studies. Our goal is to emphasize lessons learned to provide guidance about emerging strategies of Notch-based therapeutics that could be deployed safely and efficiently in patients with immune and inflammatory disorders.

## Introduction

Inflammation is a dynamic process mobilizing multiple cell types and mediators in response to stimuli that are perceived as harmful. The Notch signaling pathway is emerging as a critical regulator of inflammation, with pathogenic roles in several inflammatory and immune disorders including autoimmunity and allergic airway inflammation. In addition, Notch critically regulates graft-versus-host disease (GVHD) and graft rejection, the major complications mediated by immune responses to foreign tissue antigens after life-saving transplantation procedures, such as transplantation of allogeneic bone marrow or solid organ allografts. Therapeutic strategies to inhibit Notch signaling have first been developed preclinically and in early phase clinical trials to target oncogenic functions of the Notch pathway in tumor cells, or in tumor angiogenesis. However, many of these strategies are now also actively investigated in preclinical settings for their therapeutic value in non-malignant inflammatory disorders.

In this review, we discuss emerging concepts about the effects of Notch signaling in the regulation of mature immune cell function, beyond the role of the Notch pathway that was first established in immune cell development (Osborne and Minter, 2007; Radtke et al., 2010; Yuan et al., 2010; Brandstadter and Maillard, 2019). First, we outline unique molecular features of the Notch pathway that underlie the most promising therapeutic strategies to inhibit Notch activation. We discuss the oncogenic functions of Notch signaling that first inspired an intense interest in therapeutic targeting of Notch signaling, with a special focus on lessons learned from the successes and challenges of preclinical and early clinical investigations of Notch inhibition in cancer. We then review a growing body of work uncovering profound effects of Notch signaling in non-malignant inflammatory disorders, including immune disorders with high relevance to human disease. Specific Notch ligands and receptors play dominant functions in the interaction of immune cells with their microenvironment, opening therapeutic perspectives based on their transient targeted inhibition at sensitive stages of immune cell differentiation and function. Integrating lessons learned in cancer therapeutics and in preclinical studies of Notch signaling in the immune system, we will discuss emerging concepts that could pave the way toward effective development of Notch-based therapeutic strategies in non-malignant inflammatory disorders.

## Mechanisms and Function of Notch Signaling

**FIGURE 1:**
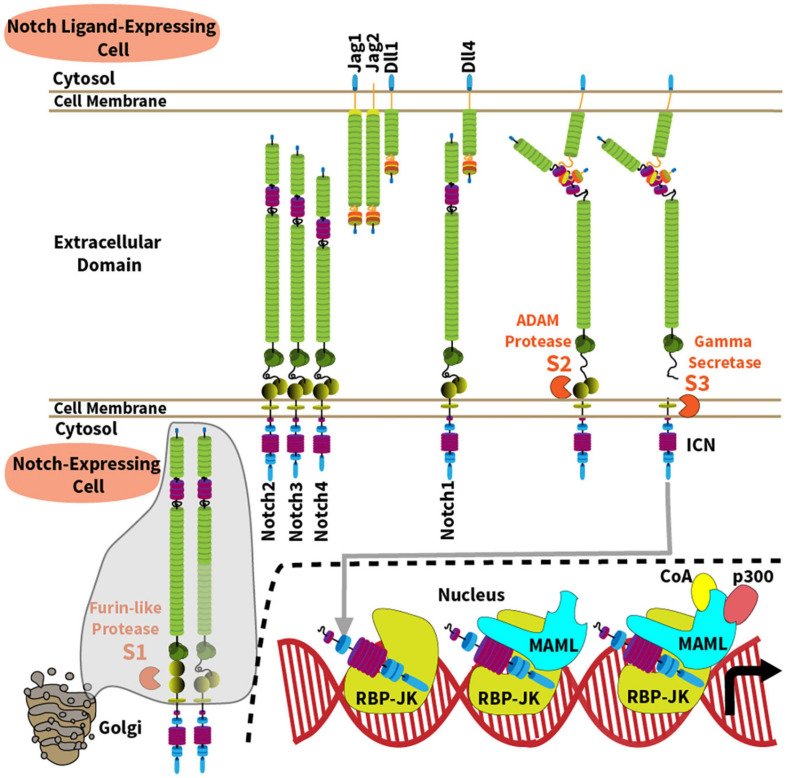
Overview of the Notch signaling pathway. The Notch signaling pathway operates between four cell surface Notch receptors (Notch1-4) and four agonistic Notch ligands from the Jagged (Jag1, Jag2) and Delta-like families (Dll1, Dll4). Mechanisms of Notch activation and canonical signaling are depicted along the following steps: (1) A furin-like protease cleaves the Notch receptor into a transmembrane heterodimer during its transit to cell surface through the Golgi complex (S1 site); (2) Ligand-receptor binding generates a physical force onto the extracellular domain of the Notch receptor, allowing ADAM10-mediated proteolysis at the S2 site which is normally hidden within a “negative regulatory region” of the receptor; (3) ADAM10 generates a membrane-bound intermediate that becomes rapidly sensitive to intramembrane proteolysis by the γ-secretase complex (S3 site). As a result, intracellular Notch (ICN) is released into the cytoplasm and translocates into the nucleus. (4) ICN binds with the DNA-binding protein RBP-Jκ (also known as CSL); (5) ICN and RBP-Jκ recruit a member of the Mastermind-like (MAML) family of transcriptional coactivators via the N-terminal MAML alpha-helical domain; (6) In turn, MAML proteins recruit other transcriptional co-activators (CoA) and p300, respectively, to enhance transcription of Notch target genes.

A Notch-related phenotype was first described by John Dexter and Thomas Morgan more than a century ago, based on inherited changes that looked like “notches” at the wing margin of *Drosophila melanogaster* fruit flies (Dexter, 1914; Morgan, 1917). Since the fly *Notch* gene was cloned in 1983, the Notch signaling pathway has emerged as an essential evolutionarily conserved pathway for the development of all metazoans (Artavanis-Tsakonas et al., 1983). Notch is important for directing cell-to-cell communication and cell fate decisions throughout embryogenesis and into postnatal life, where Notch helps maintain homeostasis of selected tissues. However, Notch has unique characteristics that distinguish it from other evolutionarily conserved pathways. First, activation of Notch signaling is enacted in *trans* between adjacent cells (Figure 1). Second, canonical Notch signaling does not rely on signal amplification from second messengers to enact its functions, because its cleaved intracellular domain can translocate to the nucleus in order to stimulate gene transcription, before being rapidly degraded (Kopan and Ilagan, 2009; Kovall et al., 2017). These features enable careful temporal and spatial regulation of Notch signaling intensity.

In mammals, five Notch ligands interact with four Notch receptors (Notch1-4), referred to as Notch ligand or receptor “paralogs” (Ellisen et al., 1991; Weinmaster et al., 1992; del Amo et al., 1993; Lardelli et al., 1994; Uyttendaele et al., 1996). Agonistic Notch ligands belong to the Delta-like (Dll1, Dll4) and the Jagged family (Jag1, Jag2) (Figure 1), while Dll3 functions as a natural antagonist. Although Notch ligands are present in multiple tissues, their spatially restricted expression in defined cellular niches represents a recurrent mode of Notch signaling regulation. Notch1 and Notch2 are expressed by a wide range of cell types. Notch3 is primarily found in developing T cells, vascular smooth muscle and pericytes, and Notch4 in the endothelium. However, recent work reported new functional roles for Notch3 and Notch4 in other cells relevant to immune disorders such as synovial fibroblasts and macrophages for Notch3, and regulatory T cells for Notch4 (Harb et al., 2020; Lopez-Lopez et al., 2020; Wei et al., 2020). Notch receptors are single-pass transmembrane proteins with variations primarily in their extracellular ligand binding and intracellular signaling domains (Figure 1). Prior to ligand-receptor binding, the immature Notch receptor is cleaved in the Golgi complex by a furin-like convertase on its way to the cell surface. Furin-like convertases cleave Notch proteins at their S1 site to generate a non-covalently bound heterodimer. The joining ends of this heterodimer form a S2 region that is normally hidden within a “negative regulatory region,” before it becomes vulnerable to cleavage by ADAM family metalloproteases. Ligand-receptor binding generates a physical force pulling on the Notch receptor, which exposes the S2 site to proteolytic cleavage by an ADAM metalloprotease (mostly ADAM10). S2 cleavage then generates a transmembrane intermediated that becomes sensitive to proteolysis by the γ-secretase complex at a third intramembrane site (S3) (De Strooper et al., 1999; Wolfe, 2020). γ-secretase releases the intracellular domain of Notch (ICN), allowing it to translocate into the nucleus to form a nuclear complex with the DNA-binding transcription factor RBP-Jκ, also known as CBF1/Suppressor-of-hairless/Lag-1 (CSL) (Tamura et al., 1995). The ICN/RBP-Jκ complex then enhances transcription of target genes through association with a Mastermind-like family coactivator (MAML1-3) and other proteins as part of a large transcriptional activation complex (Petcherski and Kimble, 2000; Wu et al., 2000; Nam et al., 2006; Wilson and Kovall, 2006). Importantly, transcriptional targets of Notch signaling are context-specific, and many reside in tissue-specific enhancers, enabling versatile functional outputs. In addition, non-canonical pathways of Notch signaling that bypass RBP-Jκ and MAML have also been reported in immune cells, although their mechanisms and relative importance remain debated (Shin et al., 2006, 2014; Auderset et al., 2012; Dongre et al., 2014; Charbonnier et al., 2015; Harb et al., 2020).

**FIGURE 2:**
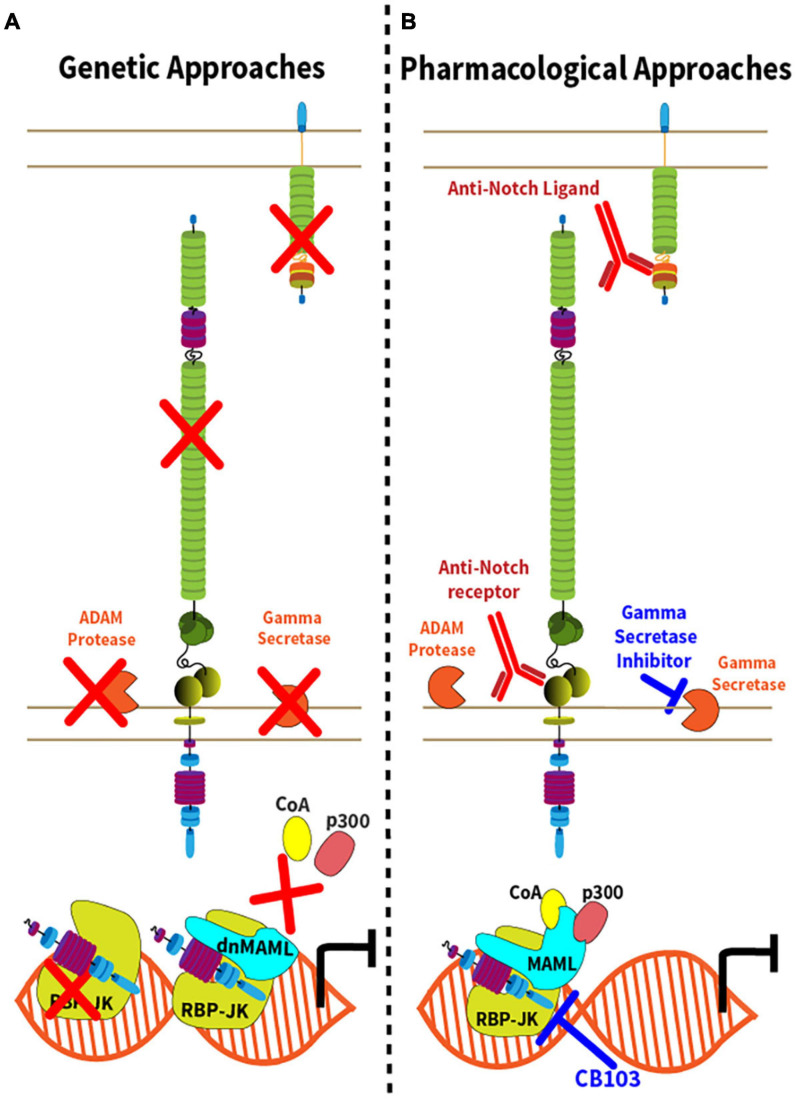
Genetic and pharmacological approaches to Notch inhibition. **(A)** Genetic inactivation strategies leading to inhibition of the Notch signaling pathway are represented by red “X” with the exception of dnMAML, where the red “X” depicts the disruption of the Notch transcription activation complex due to expression of a truncated, dominant negative (dn) form of MAML1. In addition to conditional expression of dnMAML, commonly used approaches include conditional inactivation of Notch ligand genes, Notch receptor genes, *Adam10*, genes encoding components of the γ-secretase complex or *Rbpj*; **(B)** Strategies of pharmacological inhibition of Notch signaling either though the administration monoclonal antibodies targeting the Notch ligands or receptors, or small molecule inhibitors. γ-secretase inhibitors target components of the γ-secretase complex. CB-103 inhibits the Notch transcription complex.

The unique mechanisms of Notch activation have inspired genetic and pharmacological strategies of Notch inhibition (Figure 2 and Table 1). These loss-of-function strategies are essential to rigorously evaluate the effects of the Notch pathway *in vivo*, and some have translational potential. Conditional inactivation of Notch ligand and receptor genes in specific cell types requires knowledge of the expression pattern and relative importance of Notch ligand and receptor paralogs (Figure 2A). For example, combined *Notch1* and *Notch2* inactivation accounts for most effects of Notch signaling in mature T cells, although *Notch1* loss is dominant in some contexts and *Notch4* inactivation was also reported to affect Tregs (Auderset et al., 2012; Roderick et al., 2013; Tran et al., 2013; Backer et al., 2014; Charbonnier et al., 2015; Wei et al., 2020). In contrast, *Notch1* inactivation alone blocks the effects of the pathway in early T cell development, while *Notch2* loss is sufficient to inhibit Notch signaling in mature B cells and in dendritic cells (Radtke et al., 1999; Saito et al., 2003; Lewis et al., 2011). For Notch ligands, initial studies have focused on the role of their expression in professional antigen-presenting cells, such as conventional dendritic cells (Amsen et al., 2004). However, recent work highlighted critical immunological roles for Dll1 and Dll4 Notch ligands expressed by non-hematopoietic fibroblastic stromal cell niches in secondary lymphoid organs (Fasnacht et al., 2014; Chung et al., 2017; Perkey et al., 2020). Other genetic strategies that block canonical Notch signaling include inactivation of *Rbpj*, encoding RBP-Jκ, and conditional expression of dnMAML, a truncated N-terminal fragment of Mastermind-like1 (MAML1) fused to GFP that exerts potent dominant negative activity downstream of all Notch receptors (Tanigaki et al., 2002; Maillard et al., 2004). Although Notch-unrelated functions of MAML proteins have been reported, dnMAML only contains ca. 60 amino acids from the N-terminal MAML1 alpha-helix that bind ICN and RBP-Jκ, but not any other known partners. Thus, all effects reported so far for dnMAML have been related to its impact on Notch signaling. Finally, Notch loss-of-function phenotypes can also be induced by targeting other essential genes for Notch activation, e.g., *Mib1* (encoding Mind bomb 1, an ubiquitin ligase critical in Notch ligand-expressing cells); *Pofut1* (encoding an *O*-Fucosyltransferase essential to modify mature Notch receptors); *Adam10* (encoding the ADAM10 metalloprotease); and genes encoding subunits of the γ-secretase complex (such as *Psen1/2*).

**TABLE 1:** List of key Notch inhibitors tested preclinically or clinically so far, subcategorized by name/alias, their target and cross-reactivity to humans (h), mice (m), or primates (p).

Drug/Alias name	Target	Latest phase, indication	Key references	Clinical Trials Identifier
DAPT	γ-secretase	Preclinical: Tumor	Morohashi et al., 2006	
MRK-560	γ-secretase- Presenilin1	Preclinical – T-ALL	Best et al., 2007; Habets et al., 2019	
MRK-003	γ-secretase	Preclinical – T-ALL, solid tumor	Ramakrishnan et al., 2012; Tanaka et al., 2015	
LY900009	γ-secretase	Phase I: Tumor	Pant et al., 2016	NCT01158404
AL 101 (BMS-906024)	γ-secretase	Phase II: Tumor; Preclinical: Insulin resistance	Ferrarotto et al., 2019; Sparling et al., 2020	NCT04461600 NCT03691207 NCT01292655 NCT01363817 NCT01653470
Crenigacestat (LY3039478)	γ-secretase	Phase II: Tumor	Mancarella et al., 2020	NCT02836600 NCT02906618 NCT02917733 NCT02659865 NCT02518113 NCT02784795 NCT01695005 NCT03502577
MK0752	γ-secretase	Phase II: Tumor	Whitehead et al., 2012	NCT00756717 NCT00803894 NCT00572182 NCT00645333 NCT01098344 NCT01295632 NCT01243762 NCT00106145 NCT00100152
Nirogacestat (PF-03084014)	γ-secretase	Phase III: Tumor	Kummar et al., 2015	NCT02299635 NCT02462707 NCT02338531 NCT01981551 NCT01876251 NCT02955446 NCT02109445 NCT00878189 NCT04195399 NCT03785964
RO4929097 (RG473)	γ-secretase	Phase II: Tumor	Luistro et al., 2009; Sahebjam et al., 2013; Lee et al., 2015	NCT01238133 NCT01175343 NCT01154452 NCT01198535 NCT01232829 NCT01141569 NCT01122901 NCT01116687 NCT01131234 NCT01120275 NCT01217411 NCT01193881 NCT01193868 NCT01269411 NCT01088763 NCT01251172 NCT01216787 NCT01145456 NCT01158274 NCT01071564 NCT01270438 NCT01151449 NCT01196416 NCT01096355 NCT01189240 NCT01192763 NCT01200810 NCT01198184 NCT01119599 NCT01149356 NCT01070927 NCT01236586 NCT01208441 NCT01218620 NCT00532090
CT16	hNotch 2/3, hEGFR	Preclinical: Tumor	Hu et al., 2017	
PTG12	hEGFR/hNotch 2/3	Preclinical: Tumor	Fu et al., 2019	
Anti-NRR1	h/mNotch 1	Preclinical: Tumor, GVHD, graft rejection	Wu et al., 2010; Tran et al., 2013; Magee et al., 2019	
Anti-NRR2	h/mNotch 2	Preclinical: Tumor, GVHD	Wu et al., 2010; Tran et al., 2013	
Brontictuzumab (OMP-52M51)	hNotch 1	Phase I: Tumor	Ferrarotto et al., 2018	NCT01778439 NCT01703572 NCT02662608 NCT03031691
Tarextumab (OMP-59R5)	hNotch 2/3	Phase II: Tumor	Hu et al., 2019	NCT01277146 NCT01647828 NCT01859741
15D11	hJag 1	Preclinical: Tumor	Zheng et al., 2017	
Anti-Jag1/2	hJag1/2	Preclinical: Airway	Yan et al., 2010	
Anti-Dll1	h/mDll1	Preclinical: GVHD, graft rejection	Liang et al., 2006; Tran et al., 2013	
YW152F	h/mDll4	Preclinical: Tumors, GVHD, graft rejection	Liang et al., 2006; Ridgway et al., 2006; Tran et al., 2013; Wood et al., 2015	
MMGZ01	hDll4	Preclinical: Tumor	Jia et al., 2016; Xu et al., 2016	
mABL001	mDll4, mVEGF	Preclinical: Tumor	Kim et al., 2020	
HMD4-2	h/mDll4	Preclinical: Tumor	Yamanda et al., 2009	
Demcizumab (OMP-21M18)	hDll4	Phase I: Tumor	Smith et al., 2014; McKeage et al., 2018	NCT00744562 NCT01189942 NCT01189968 NCT01189929 NCT02722954 NCT01952249
Enoticumab (REGN421)	hDll4	Phase I: Tumor	Chiorean et al., 2015	NCT00871559
MEDI0639	hDll4	Phase I: Tumor	Jenkins et al., 2012; Falchook et al., 2015	NCT01577745
Navicixizumab (OMP-305B83)	hDll4, hVEGF	Phase I: Tumor	Jimeno et al., 2019; Perez-Fidalgo et al., 2020	NCT03035253 NCT02298387 NCT03030287
ABT-165	hDll4, hVEGF	Phase II: Tumor	Li et al., 2018; Wainberg et al., 2018	NCT03368859 NCT01946074
NOV1501 (ABL001; HD105)	h/pDll4, h/pVEGF	Phase II: Tumor	Choi et al., 2016; Lee et al., 2016; Sosa Iglesias et al., 2018; Kim et al., 2020; Yeom et al., 2021	NCT03292783 NCT04492033
IMR-1	hRBPJ/ICN1/MAML	Preclinical: Tumor	Lafkas et al., 2015	
RIN1	hRPBJ	Preclinical: Tumor	Hurtado et al., 2019	
SAHM1	h/mICN1/RBPJ	Preclinical: Tumor, Airway	Moellering et al., 2009; KleinJan et al., 2018; Takam Kamga et al., 2019	
CB-103	hRBPJ/MAML	Phase I/IIa	Lehal et al., 2020	NCT04714619 NCT03422679

*Note that many of the drugs reactive to human targets may also be active in primates. Cross-reactivities in this table are only listed if a primary source of literature could be found. Clinical trials using these drugs are listed with their unique identifier in clinicaltrials.gov.*

Complementing genetic approaches, pharmacological strategies with translational potential are depicted in Figure 2B. γ-secretase inhibitors (GSI) block the rate-limiting step of ligand-regulated proteolytic activation for all Notch receptors, thus achieving systemic pan-Notch inhibition (De Strooper et al., 1999; Wolfe, 2020). Monoclonal antibodies have been developed to inhibit individual Notch ligands or receptors, both in mice and in humans (Noguera-Troise et al., 2006; Ridgway et al., 2006; Wu et al., 2010; Tran et al., 2013; Choy et al., 2017). By blocking specific Notch ligand and receptor paralogs, these antibodies have the potential to bypass some of the systemic effects of pan-Notch inhibition, thus increasing the therapeutic window. Finally, recent work reported the discovery of CB-103, a new orally active small molecule inhibitor of the Notch transcription activation complex, providing an alternative path to therapeutic Notch inhibition (Lehal et al., 2020). To facilitate understanding of currently available therapeutic interventions, we provide a list of key existing reagents together with their most advanced stage of development to date (Table 1). Importantly, findings in immune and inflammatory disorders remain preclinical, while our understanding of Notch targeting in cancer relies on both preclinical and clinical observations.

## Targeting Notch Signaling in Cancer and in Tumor Angiogenesis

The oncogenic role of Notch signaling in cancer first sparked interest in therapeutic targeting of the Notch pathway, inspiring a first generation of preclinical and early clinical studies. Oncogenic activation of Notch signaling was originally described in 1991 in T cell acute lymphoblastic leukemia (T-ALL) based on a rare t(7;9) chromosomal translocation forcing expression of a constitutively active form of NOTCH1 (Ellisen et al., 1991). Later investigations showed that > 50% of T-ALL patients harbored *NOTCH1* point mutations or other genetic events activating Notch signaling (Weng et al., 2004). While *NOTCH1* mutations allowed for ligand-independent activation, they remained sensitive to GSI-mediated inhibition and prompted preclinical studies of Notch blockade as a targeted therapy in T-ALL (Weng et al., 2006; Cullion et al., 2009; Real et al., 2009; Tatarek et al., 2011; Samon et al., 2012; Sanchez-Martin et al., 2017). Other studies revealed oncogenic Notch activation in a wider range of cancer types, including breast cancer, adenoid cystic carcinoma and a variety of B cell lymphoproliferative disorders, as reviewed in Aster et al. (2017). In some contexts, oncogenic Notch activation appeared to proceed via interaction of unmutated Notch ligands and receptors, suggesting that Notch pathway mutations may only identify a fraction of the Notch-sensitive tumors (Fabbri et al., 2017). In addition, other investigators identified a critical role of *DLL4/NOTCH1* interactions as well as a crosstalk of the Notch and VEGF pathways in tumor angiogenesis (Noguera-Troise et al., 2006; Ridgway et al., 2006). These effects of Notch in the tumor microenvironment formed the initial rationale for several drug development programs and motivated testing of anti-DLL4 antibodies in cancer patients. Importantly, most cancer-related indications of therapeutic Notch inhibition focused on continuous and long-lasting inhibition of the pathway as a desirable outcome, which also contributed to reported side effects.

### Development of γ-Secretase Inhibitors (GSIs)

The first drugs used to target Notch in the clinic originated from groundbreaking work designed to target the γ-secretase complex in Alzheimer’s disease. In Alzheimer’s, γ-secretase plays a critical role in the cleavage of the amyloid precursor protein, subsequently forming aggregates of amyloid-beta peptides in the brain as a major contributor to disease progression (Haass and Selkoe, 1993). GSIs target Presenilin components of the γ-secretase complex, locking it in a closed conformation and inhibiting the deposition of amyloid-beta peptides. This discovery inspired clinical trials of GSIs in Alzheimer’s disease. Importantly, originally designed GSIs also inhibited proteolytic activation of all transmembrane-bound Notch receptors, effectively silencing all Notch activity (De Strooper et al., 1999; Wolfe, 2020). Although GSIs have not proven successful so far in Alzheimer’s disease, they opened the door to preclinical and early clinical studies of Notch inhibition in cancer (Doody et al., 2013).

### GSIs in Human Cancer Clinical Trials

The first Phase 1 clinical trial targeting Notch evaluated the GSI MK-0752 in relapsed/refractory T-ALL (Deangelo et al., 2006). MK-0752 is a broad-spectrum GSI originally designed to treat Alzheimer’s disease, but repurposed for the treatment of T-ALL. MK-0752 demonstrated some efficacy in inhibiting T-ALL expansion and showed disease regression in a small number of patients (Deangelo et al., 2006). However, many patients suffered significant diarrhea in a dose-dependent manner, which may have limited treatment to suboptimal or intermittent dosing, thus decreasing drug efficacy. Intestinal side effects of GSIs likely resulted from on-target toxicity from pan-Notch inhibition in gut (van Es et al., 2005; Deangelo et al., 2006; VanDussen et al., 2012). Indeed, preclinical studies in mice using GSIs and/or genetic approaches showed that Notch is critical for the homeostasis of intestinal stem and progenitor cells. *Rbpj* inactivation in *Villin-Cre^+^* intestinal cells or the use of GSIs induced the differentiation of intestinal crypt cells into post-mitotic goblet cells, leading to severe diarrhea (van Es et al., 2005). Paneth cells, located at the basis of intestinal crypts, appeared to function as a niche for intestinal stem cells (Sato et al., 2011). This damage was most pronounced when both Notch1 and Notch2 were inhibited (Riccio et al., 2008; Wu et al., 2010). In studies involving irradiation, even more severe epithelial intestinal damage was documented after GSI treatment, suggesting a role for Notch in intestinal regeneration (Tran et al., 2013). Diarrhea is not exclusive to MK-0752. Other first-generation GSIs such as AL-101, Crenigacestat, and Nirogacestat also exhibited similar gastrointestinal toxicities, along with a few other complications such as fatigue, anemia, thrombocytopenia, and hypophosphatemia that could also have been related to the concomitant use of chemotherapy (Messersmith et al., 2015; Cook et al., 2018; El-Khoueiry et al., 2018; Massard et al., 2018). Nonetheless, the use of first-generation GSIs has not been completely discontinued in clinical trials. In some ongoing trials, first-generation GSIs are being evaluated for their use in combination therapies with already approved anti-cancer treatment modalities. These studies deploy GSIs at tolerable doses or with decreased frequency, while working synergistically with other treatments to suppress tumor growth. In preclinical data, intermittent GSI dosing indeed appeared to open a therapeutic window (Cullion et al., 2009; Tatarek et al., 2011). Another approach relies on the combination of GSIs and corticosteroids, which appear to suppress the gastrointestinal side effects of pan-Notch inhibition (Real et al., 2009; Samon et al., 2012). Finally, selection of GSI-resistant tumor cells represents another barrier to therapeutic Notch inhibition (Palomero et al., 2007; Knoechel et al., 2014; Aster et al., 2017). Altogether, first-generation GSIs have not been tolerated well enough to show deep efficacy as stand-alone drugs. Several strategies are being tested or considered to improve the therapeutic index of these drugs: (1) Modified administration schedules, for example with intermittent administration; (2) Combination therapy with other drugs to enhance efficacy or mitigate toxicity; (3) Development of new generations of drugs that show enhanced specificity for Notch signaling in cancer (Habets et al., 2019; Yang et al., 2020). Importantly, these changes have to be paired with a better identification of patients with documented Notch tumor activation, as opposed to unselected cancer patients.

### Monoclonal Antibodies Blocking Notch Receptors and Ligands

To bypass the toxicities of systemic pan-Notch inhibition, humanized monoclonal antibodies have been developed against individual Notch1, Notch2, and Notch3 paralogs. In mice, Notch1 inhibition alone was much better tolerated than combined Notch1/2 inhibition, consistent with the redundant role of Notch1 and Notch2 in intestinal stem cell homeostasis (Riccio et al., 2008; Wu et al., 2010). In preclinical models, Notch1 or Notch3 inhibition alone had promising activity in specific models of T-ALL and breast cancer (Wu et al., 2010; Choy et al., 2017). To date, blocking antibodies against Notch1 and Notch2/3 have been clinically evaluated for the treatment of tumors (Smith et al., 2014; Yen et al., 2015; Ferrarotto et al., 2018). Reports on anti-Notch1 blockade indicated some activity in patients with hematological and solid tumors (Casulo et al., 2016; Ferrarotto et al., 2018). However, despite indications in mice, gut toxicity was still a problem in phase 1 trials (Casulo et al., 2016; Ferrarotto et al., 2018). Initial data of anti-Notch2/3 blocking antibodies were promising in solid tumors (Yen et al., 2015). However, a randomized phase 2 trial showed that benefits were contributed primarily by chemotherapy and not anti-Notch2/3 (Pietanza et al., 2015; Hu et al., 2019). Altogether, these findings represent progress in limiting the systemic toxicity of Notch inhibition, although clinical activity was not impressive so far, at least when patients were not selected upfront for having Notch-dependent tumors.

Dll4-regulated tumor angiogenesis has inspired the development of anti-DLL4 antibodies, as well as their study in both preclinical and early clinical models. Solid tumors induce a network of blood vessels to provide continuous oxygen and nutrient supplies that sustain their growth (Folkman, 1971). To date, vascular endothelial growth factor (VEGF) remains the primary clinical target used to suppress tumor angiogenesis (Keck et al., 1989; Leung et al., 1989). The role of Notch in angiogenesis has been extensively documented as part of a crosstalk between DLL4/NOTCH1 and VEGF (Noguera-Troise et al., 2006; Ridgway et al., 2006). Preclinical work showed that the effects of anti-DLL4 and anti-VEGF do not overlap, with a potential for anti-DLL4 to overcome resistance to anti-VEGF therapy (Ridgway et al., 2006). Mechanistically, DLL4 inhibition increased angiogenic sprouting, but led to non-productive blood vessel formation and diminished tumor growth (Noguera-Troise et al., 2006). In a human phase I clinical trial of DLL4 blockade, intestinal side effects were not observed, but cardiovascular events associated with prolonged DLL4 inhibition were reported, including hypertension, pulmonary hypertension and congestive heart failure (Smith et al., 2014; Chiorean et al., 2015; Falchook et al., 2015; McKeage et al., 2018). In addition, chronic DLL4 blockade has been reported to cause vascular anomalies in rats and monkeys (Yan et al., 2010). Thus, while anti-DLL4 may bypass the side effects previously observed with GSI or combined anti-NOTCH1/2 antibodies, chronic DLL4 inhibition as required for optimal anti-cancer activity remains problematic.

### New Pharmacological Approaches to Target Notch Signaling in Cancer

New generation GSIs are being developed to target specific components of the γ-secretase complex (Churcher et al., 2006; Best et al., 2007; Habets et al., 2019). MRK-560 has gained traction as a treatment for T-ALL because studies have shown MRK-560 to be more tolerable, with reduced gastrointestinal side effects in preclinical animal models (Habets et al., 2019). Different γ-secretase complexes contain variable proportions of the two Presenilins, PSEN1 and PSEN2 (Kimberly et al., 2003). MRK-560 has a ca. 100-fold selectivity for PSEN1 over PSEN2 (Borgegard et al., 2012). In contrast to dominant expression of PSEN1 in T-ALL, PSEN1 and PSEN2 both expressed in mouse and human intestine (Habets et al., 2019). Indeed, pharmacological PSEN1 inhibition by MRK-560 attenuated T-ALL growth in mice (Habets et al., 2019). As a separate approach, Lehal et al. (2020) recently reported the development of CB-103, a first-in-class inhibitor of the Notch transcription complex. CB-103 showed promising preclinical activity in the treatment of T-ALL and other Notch-dependent tumors, including GSI-resistant cell lines, without inducing significant intestinal toxicity for reasons that remain to be fully clarified. This promising activity profile has led to ongoing clinical trials of CB-103 in cancer. Thus, new compounds are being developed to target Notch signaling in cancer, and it will be interesting to evaluate if they also have therapeutic potential in non-malignant conditions such as Notch-driven immune disorders.

## Targeting Notch Signaling in Inflammatory and Immune Disorders

In the immune system, Notch signaling was first identified for its essential role in early T cell development (Pui et al., 1999; Radtke et al., 1999). *Dll4* inactivation in cortical thymic epithelial cells or *Notch1* loss in lymphoid progenitors blocks T cell development in the thymus (Radtke et al., 1999; Hozumi et al., 2008; Koch et al., 2008). Other developmental functions of Notch signaling regulate the emergence of definitive hematopoietic stem cells in the fetus as well as various aspects of B cell, innate lymphoid cell and dendritic cell development (Tanigaki et al., 2002; Saito et al., 2003; Lewis et al., 2011; Satpathy et al., 2013; Yang et al., 2013). Beyond development, mature immune cells express Notch receptors (most commonly a combination of Notch1 and/or Notch2), and they can interact with Notch ligands in their microenvironment (e.g., in secondary lymphoid organs or in tissues). On this basis, Notch signaling exerts essential functions during specific immune responses, including in many contexts with relevance to human health and disease. These discoveries have identified a new set of Notch-related therapeutic opportunities.

From a translational perspective, the principles of targeting Notch signaling in immune and inflammatory disorders build on different rules than in cancer-related indications. In cancer, prolonged Notch inhibition is desirable, which has been linked to the occurrence of problematic on-target side effects. In addition, cancer cells can be selected for acquired resistance to Notch inhibition through epigenetic and other mechanisms. In the immune system, selection of clones resistant to Notch inhibition is not an issue, and Notch blockade can be applied transiently at sensitive stages of immune cell differentiation and function. This strategy would have the advantage to preserve Notch-mediated functions in lymphoid development and beneficial immune responses that develop outside of the transient windows of Notch inhibition. We will now review emerging evidence about essential pathogenic functions of Notch signaling in immune and inflammatory disorders that could become the target of therapeutic interventions.

### Experimental Autoimmune Encephalomyelitis and Multiple Sclerosis

Minter and collaborators first described a pathogenic role of Notch signaling in T cells mediating Experimental Autoimmune Encephalomyelitis (EAE) in mice, a model that shares many features with human Multiple Sclerosis (MS) (Minter et al., 2005). Using GSIs *in vivo* and *ex vivo*, the authors reported attenuated EAE severity and a decreased propensity of CD4^+^ T cells to differentiate into Th1 cells expressing *Tbx21* (encoding the master transcription factor T-bet). These findings were consistent with critical effects of Notch signaling in CD4^+^ T cells, which play a key role in EAE pathogenesis both through Th1 and Th17 differentiation. The T cell-intrinsic pathogenic functions of Notch during EAE were subsequently confirmed via T cell-specific *Rbpj* inactivation or expression of the pan-Notch inhibitor DNMAML, which provided high protection from EAE induced by polyclonal T cells or by myelin-specific 2D2 T cell receptor transgenic T cells (Sandy et al., 2013b). The impact of RBP-Jk and DNMAML was consistent with a dominant role of canonical RBP-Jk/MAML-dependent signaling in encephalitogenic T cells during EAE. In this study, Notch inhibition did not impair Th1 and Th17 differentiation in the periphery, but profoundly inhibited the accumulation of T cells in the brain and spinal cord. Other investigators reported protection from EAE upon systemic inhibition of Dll4 Notch ligands, with an impact on T cell infiltration in the central nervous system, Treg expansion and Th1/Th17 differentiation, respectively (Bassil et al., 2011; Reynolds et al., 2011; Eixarch et al., 2013). Finally, Notch may also impact Th9 differentiation and myelin repair mechanisms (Jurynczyk et al., 2005; Elyaman et al., 2012).

Altogether, Notch signaling is emerging as a central regulator of EAE pathogenesis, with T cell-intrinsic functions playing a major role. Additional investigations combining genetic and pharmacological approaches are needed to carefully dissect the impact of Notch signaling in T cells vs. other cell types involved in EAE progression. From a translational perspective, it will be essential to evaluate the role of Dll4 as opposed to other Notch ligands; map the cellular source of Notch ligands as well as their spatial and temporal interactions with T cells; and define the critical time windows during which systemic Notch inhibition induces maximum benefits. For example, it would be interesting to define if and when transient systemic Notch ligand inhibition can abort disease flares by itself or in combination with other interventions.

### Graft-Versus-Host Disease After Allogeneic Hematopoietic Cell Transplantation

Allogeneic bone marrow or hematopoietic cell transplantation (allo-HCT) is a potentially life-saving therapeutic modality for patients with benign and malignant hematological disorders, including leukemias and lymphomas. However, acute and chronic graft-versus-host disease (GVHD) remain major immune complications of the procedure that limit its success and curtail its widespread use. GVHD is triggered by donor-derived T cells in the hematopoietic graft that recognize foreign tissue antigens in the recipient, leading to target organ damage. Unmet clinical needs include the occurrence of GVHD in a high fraction of patients despite universal use of prophylactic immunosuppression; severe acute GVHD, especially when resistant to corticosteroids; extensive forms of chronic GVHD, which induce serious life-long morbidity; and the need to control GVHD without eliminating the beneficial graft-versus-tumor effects of allo-HCT in cancer patients.

In the past 10 years, Notch inhibition in donor T cells has emerged as an attractive new strategy to control GVHD without inducing global immunosuppression. Protective effects of Notch inhibition were observed in multiple mouse allo-HCT models of acute and chronic GVHD, across major and minor histocompatibility antigen mismatches, after conditioning regimens of variable types and intensity, and also in a model of aplastic anemia mediated by alloreactive T cells (Zhang et al., 2011; Mochizuki et al., 2013; Roderick et al., 2013; Sandy et al., 2013a; Tran et al., 2013). Inhibition of canonical Notch signaling accounted for all major effects of Notch blockade in T cells (Zhang et al., 2011; Tran et al., 2013; Charbonnier et al., 2015). Combined Notch1 and Notch2 blockade in T cells was necessary to achieve maximum effects of Notch inhibition, but with a major role for Notch1 (Tran et al., 2013; Radojcic et al., 2018). In one report, protective effects were reported upon *Notch1/2* inactivation only in regulatory T cells (Tregs) (Charbonnier et al., 2015). In terms of Notch ligands, Dll1 and Dll4 in the host accounted for all the effects of Notch signaling in GVHD, with a dominant role for Dll4 (Mochizuki et al., 2013; Tran et al., 2013; Chung et al., 2019; Perkey et al., 2020). Mechanistically, Notch inhibition blunted the production of multiple inflammatory cytokines in alloreactive T cells, including IFNγ, TNFα and IL-17, while leading to increased expansion of preexisting Tregs, enhanced Treg function and decreased accumulation of T cells in the gut (Zhang et al., 2011; Sandy et al., 2013a; Tran et al., 2013; Charbonnier et al., 2015). Notch inhibition rapidly established a unique transcriptional signature in alloreactive T cells within days after transplantation, although all direct transcriptional targets remain to be identified systematically through genome-wide approaches (Chung et al., 2019). Importantly, and unlike conventional immunosuppression, Notch blockade did not inhibit the activation and expansion of alloreactive T cells in secondary lymphoid organs, and it preserved high levels of cytotoxicity and anti-cancer activity (Zhang et al., 2011; Sandy et al., 2013a; Tran et al., 2013). Thus, Notch inhibition in T cells did not cause global immunosuppression, but instead induced a beneficial pattern of immunomodulation after allo-HCT.

From a translational perspective, systemic pan-Notch inhibition with GSIs showed on-target activity in alloreactive T cells similar to that of genetic interventions, but also side effects in the gut that were poorly tolerated (Tran et al., 2013). Although not unexpected given the known functions of Notch signaling in intestinal epithelial cells during homeostasis, this on-target toxicity was enhanced after transplantation, likely due to a role of Notch signaling in intestinal regeneration after injury from conditioning irradiation (van Es et al., 2005; Riccio et al., 2008; Tran et al., 2013). Thus, GSIs are not promising in this context. Bypassing these limitations, targeted antibody-mediated inhibition of the Notch ligands Dll1 and Dll4 were efficient at controlling GVHD and well tolerated (Tran et al., 2013; Chung et al., 2017). Importantly, early Dll1/4 inhibition during the first 2 days after allo-HCT proved essential to induce GVHD protection, suggesting that critical pathogenic Notch signals are delivered to incoming T cells very early after transplantation (Chung et al., 2017). Conversely, short-term Notch inhibition even with a single dose of anti-Dll1/4 antibodies was sufficient to confer long-term protection from GVHD. During early days after allo-HCT, T cells were found to interact with Dll1/4 ligands expressed by specialized niches of non-hematopoietic fibroblastic reticular cells lineage traced with a *Ccl19-Cre* transgene in secondary lymphoid organs (Chung et al., 2017; Perkey et al., 2020). The dominant role of non-hematopoietic cells as a source of Notch ligands in this context came as a surprise, as dendritic cells as well as other professional antigen-presenting cells had been considered previously as the likely source of ligands. Nevertheless, this pattern of Notch ligand-receptor interactions in secondary lymphoid organs is reminiscent of regulation in the thymus, where Notch ligands in non-hematopoietic thymic epithelial cells interact with Notch receptors in T cell progenitors.

Altogether, these findings support continued translational investigations of Notch ligand inhibition as a new strategy to prevent GVHD. The long-term benefits of short-term Notch inhibition at the time of transplant are particularly relevant, as they avoid the consequences of prolonged Notch blockade, including inhibition of Dll4/Notch1-driven T cell development in the thymus and other potential negative consequences. Importantly, emerging data indicate that the central role of Notch signaling in GVHD is conserved from mice to non-human primates. Indeed, short-term DLL4 blockade at the time of transplant induced significant protection from GVHD in a Rhesus macaque model similar to human transplantation, even with a single dose of antibodies (Tkachev et al., 2018). Thus, Notch ligand blockade could be considered to prevent GVHD in human allo-HCT.

### Rejection After Allogeneic Solid Organ Transplantation

Acute and chronic immune-mediated rejection limit the success of solid organ transplantation in patients, such as heart, lung, liver or kidney transplant recipients. In addition, the risk of rejection mandates long-term administration of global immunosuppressive drugs, such as calcineurin inhibitors, which carry significant side effects including vascular and renal toxicity, a propensity to opportunistic infections and post-transplant lymphoproliferative disorders. Thus, new strategies to prevent rejection are needed, ideally by inducing tolerance to transplanted allogeneic organs.

Early work using *ex vivo* and *in vivo* exposure of T cells to overexpressed Notch ligands suggested a role for Notch signaling in tolerance induction (Vigouroux et al., 2003; Wong et al., 2003; Yvon et al., 2003). However, artificial features of these experimental systems did not allow for definitive conclusions about the role of Notch signaling, and instead multiple convergent reports have now identified Notch as a major pro-inflammatory pathway driving organ rejection *in vivo*. Riella et al. (2011) first reported a role for the Notch pathway in transplant rejection by targeting the Notch ligand Dll1 with monoclonal antibodies in a mouse model of heart transplantation. Together with B7-CD28 blockade, anti-Dll1 antibodies induced significant, although relatively modest, protection from heart rejection, which was associated with STAT6-dependent Th2 polarization. Conversely, Jagged2-mediated agonism accelerated rejection through an IL6-dependent pathway (Riella et al., 2013). Although these studies captured consistent pro-rejection effects of Notch signaling, they only investigated the impact of isolated Notch ligands. Using expression of the pan-Notch inhibitor DNMAML in T cells, Wong et al. (2003) reported delayed rejection of mouse allogeneic heart transplants, which was most pronounced upon concomitant CD8 depletion (Wood et al., 2015). Protection was associated with decreased T cell infiltration and an increased proportion of Tregs in the graft. Furthermore, a short course of antibody-mediated Dll1/4 blockade over 10 days led to even better protection than pan-Notch inhibition in T cells, as well as to decreased alloantibody production and complement deposition in the graft (two features of chronic rejection). Thus, systemic Dll1/4 blockade may exert protective effects through its impact on both alloreactive T cells and other pathogenic cell types (e.g., B cells and plasma cells). Recently, Riella’s group reported major protective effects of anti-Notch1 neutralizing antibodies when administered during 10 days after transplantation of MHC-mismatched heart allografts (Magee et al., 2019). Protection was associated with evidence of increased Treg expansion and function. Prolonged graft survival was particularly impressive when anti-Notch1 antibodies were combined with CTLA4-Ig, suggesting that a tolerance-like state can be achieved in these conditions.

Together, this growing body of work identifies strategies of Notch inhibition with translational potential in the prevention of organ rejection. Selective inhibition of individual Notch ligands or receptors is attractive to prevent the systemic side effects of pan-Notch inhibition, especially when applied transiently. As seen in GVHD, short-term inhibition in the peri-transplant period exerts long-term protective effects, which limits the potential consequence of prolonged Notch ligand or receptor inhibition. More work needs to identify all target cell types, and the most promising treatment combinations, although the joint effects of Notch blockade and CTLA4-Ig are particularly interesting.

### Asthma and Allergic Airway Inflammation

Asthma is characterized by bronchial hyperreactivity and airway infiltration by T lymphocytes, innate lymphoid cells, macrophages, neutrophils, mast cells and eosinophils, with important inflammatory roles for both innate and adaptive immune cells. CD4^+^ T cell differentiation to a T helper 2 (Th2) phenotype under the control of the master transcription factor GATA3 is central to disease pathogenesis via secretion of Th2 cytokines (e.g., IL-4, IL-5, IL-13), although Th17 differentiation also takes place. *Ex vivo* work using coculture with antigen-presenting cells first identified the potential for Jagged ligands to drive CD4^+^ Th2 polarization (Amsen et al., 2004). In several mouse models of Th2 differentiation, Notch was reported to directly regulate *Gata3* and *Il4* transcription, the latter through ICN binding at the *Il4* CNS2 enhancer element (Tu et al., 2005; Tanaka et al., 2006; Amsen et al., 2007; Fang et al., 2007). Other groups proposed that Notch can sustain and amplify, rather than initiate, multiple types of T helper responses (Bailis et al., 2013; Laky et al., 2015). Collectively, these studies inspired researchers to evaluate pharmacological strategies of Notch inhibition in models of asthma and allergic airway inflammation. Administration of GSIs blunted disease pathogenesis and Th2 differentiation in a mouse asthma model induced by ovalbumin sensitization (Kang et al., 2009). Other investigators reported an impact of GSIs on Th17 differentiation (Zhang et al., 2015). In a house dust mite model, eosinophil infiltration, Th2 differentiation and bronchial hyperreactivity were blunted by topical intratracheal administration of the stapled peptide SAHM1, which blocks Notch-mediated transcriptional activation (Moellering et al., 2009; KleinJan et al., 2018). Antibody-mediated blockade of Jagged1 and Dll4 ligands had opposite effects in an ovalbumin-driven model, with Jagged1 blockade ameliorating the disease and Dll4 inhibition worsening it, possibly via an effect on Tregs (Huang et al., 2017). Systemic Dll4 blockade also enhanced bronchial hyperresponsiveness and inflammation in mouse models of airway hyperreactivity following Respiratory Syncytial Virus infection (Schaller et al., 2007). Thus, the impact of individual Notch ligands and receptors in asthma and related conditions is profound but complex.

Recent work is expanding our understanding of Notch signaling in asthma, revealing new mechanisms and unexpected players. Tindemans et al. (2020) used a house dust mite mouse model of allergic airway inflammation to document genetically a major pathogenic role of *Notch1* and *Notch2* in T cells. Interestingly, transgenic *Gata3* expression in *Notch1/2*-deficient or *Rbpj*-deficient T cells only had a limited impact on their phenotype, suggesting Gata3-independent effects of canonical Notch signals. Instead, Notch was found to promote lymph node egress and trafficking of CD4^+^ T cells into the lung, possibly via a KLF2/S1PR1 axis. Recently, Chatila’s group described a Jagged1-Notch4 signaling axis at the core of asthma pathogenesis in mice, with correlative data suggesting its relevance to human patients (Xia et al., 2015; Harb et al., 2020). Alveolar macrophages showed increased *Jag1* expression when exposed to ultrafine ambient particles through a mechanism dependent on Aryl Hydrocarbon Receptor, in turn engaging Notch receptors in CD4^+^ cells (Xia et al., 2015). Surprisingly, the dominant Notch receptor in these studies of allergic airway inflammation proved to be Notch4, rather than Notch1/2 (Harb et al., 2020). Functional effects of Notch4 on Th2 differentiation were mediated by canonical RBP-Jκ-dependent Notch signaling, while other effects were not. In addition, Notch4 activation was found to predominate in Tregs, where it was linked to Wnt and Hippo activation, destabilization of the Treg program and a pro-inflammatory crosstalk with type 2 innate lymphoid cells. At this stage, the relative impact of Notch1/2-mediated and Notch4-mediated effects reported by different groups has not been resolved. Another interesting consideration is the emerging role of Notch signaling in cells other than CD4^+^ T lymphocytes. Roles for Notch signaling have been reported in the regulation of lung-infiltrating effector CD8^+^ T cells, lung-resident memory CD8^+^ T cells as well as innate lymphoid cell differentiation and function, all of which could impact asthma pathogenesis (Okamoto et al., 2008; Hombrink et al., 2016; Zhang et al., 2017). Finally, Notch is also emerging as a key regulator of bronchial epithelium homeostasis in health and disease, as continuous Jagged1/2-Notch2-mediated signals block the transdifferentiation of club cells into ciliated cells and basal epithelial stem/progenitor cells communicate with their secretory progeny via Notch signaling (Lafkas et al., 2015; Pardo-Saganta et al., 2015).

Altogether, these findings reveal the interesting and complex biology of Notch signaling in airway inflammation. The mechanisms of Notch action and all the cellular partners involved need to be investigated further, including immune and non-immune cell types. These considerations are especially relevant when systemic rather than cell-specific targeted genetic interventions are being considered in translational investigations.

### Other Immune and Inflammatory Disorders

Although space limitations prevent us from comprehensively including all work reported in the field, a role for Notch signaling has been suggested in other immune and inflammatory disorders, with various degrees of evidence. An interesting common denominator is that investigations of inflammatory disorders identify complex interactions of immune cells with non-hematopoietic partners in their environment, and new functions of Notch signaling in unexpected cell types.

In inflammatory aspects of atherosclerosis, Notch may be involved in the regulation of both endothelial function and infiltrating leukocytes. Endothelial Notch1 expression was suppressed in mice and humans on high fat diets, which correlated with increased atherosclerosis progression (Briot et al., 2015). In the same study, human aortic endothelial cells treated with lipids and inflammatory cytokines showed a significant decrease in Notch1 expression. These data were consistent with an anti-inflammatory role of endothelial Notch1 signaling. In contrast, other reports reported Notch-dependent induction of IL-6 expression in endothelial cells and a crosstalk with macrophages, polarizing them to an inflammatory state via Dll4-dependent signals (Pabois et al., 2014, 2016). In a LDL-deficient mouse model of atherosclerosis, *in vivo* antibody-mediated Dll4 blockade attenuated the progression of atherosclerotic plaques, as well as macrophage accumulation and M1 differentiation (Fukuda et al., 2012). More work is needed to fully understand the role of Notch signaling in atherosclerosis, which could be hindered by the lack of mouse models that fully recapitulate human disease.

In rheumatoid arthritis, early work suggested the presence of activated Notch1 receptors in synovial cells, especially in vascular and perivascular regions (Nakazawa et al., 2001; Gao et al., 2012). In a mouse model of collagen-induced arthritis, GSI administration decreased the clinical and pathological severity of joint inflammation (Park et al., 2015). In recent investigations of human synovial tissue at single cell resolution, Wei and colleagues identified an expanded population of *Notch3*-expressing sublining fibroblasts with evidence of Notch activation in rheumatoid arthritis patients (Wei et al., 2020). Single-cell transcriptomic analysis of synovial cell types suggested the existence of positional identity transmitted from the endothelium to synovial fibroblasts via Notch signaling. Indeed, organoid cultures of synovial fibroblasts and endothelial cells was consistent with a wave of Notch signaling propagated through a Jagged1/Notch3 signaling relay. These findings were reminiscent of earlier data showing a role for Notch in layering of smooth muscle cells in developing vessels, with *Jagged1* expression being induced as a Notch transcriptional target as part of a positive feedback loop (Manderfield et al., 2012). In a mouse model of antibody-mediated arthritis induced by transfer of K/BxN mouse serum, Notch3-deficient mice were resistant to arthritis induction and anti-Notch3 neutralizing antibodies blunted disease severity (with lesser effects for anti-Notch1 antibodies) (Wei et al., 2020). These exciting new data provide an entirely new perspective on disease pathogenesis and on the development of Notch-based therapeutics in autoimmune arthritis.

## Lessons and Future Directions

Much can be learned already from the rich biology of Notch signaling in cancer and inflammatory disorders, and from preclinical and early clinical investigations to target the Notch pathway therapeutically (Table 1).

In cancer, Notch behaves as an oncogenic pathway in a diverse range of tumors through gene translocations, mutational activation or natural interactions of the receptors with Notch ligands in the microenvironment. Notch also controls tumor angiogenesis via a crosstalk with the VEGF pathway. Yet, early clinical interventions attempted so far to target Notch signaling in cancer have been disappointing. Key issues include selection for resistant cells as well as on-target side effects that result from prolonged systemic inhibition of the Notch pathway or its components. On-target toxicities rooted in the physiological role of Notch in normal tissues have been dose-limiting and have prevented the deployment of maximally effective inhibition schedules, thus in turn likely contributing to decreased anti-tumor activity. Moving forward, it will be essential to better select patients with Notch-sensitive tumors and consider synergistic effects of combination therapies. In addition, all efforts need to be made to identify more specific strategies to target Notch signaling or its consequences in tumor cells while sparing or protecting normal tissues, especially since prolonged inhibition remains desirable in cancer therapy.

In contrast, the role of Notch signaling in immune and inflammatory disorders represents a more recent discovery leading to new therapeutic opportunities. Of note, all information available so far stems from preclinical disease models, thus the benefits of Notch inhibition in human inflammatory disorders remains to be established. To maximize chances of success, we believe that it will be important to consider rules that differ from those applying to Notch signaling in cancer. Prolonged Notch inhibition is not always necessary to achieve long-term therapeutic benefits in immune disorders. Instead, pulses of Notch inhibition applied at critical times in the disease course can reprogram immune cells to a less pathogenic state, or decrease immune cell trafficking to target organs, while expanding and reinforcing the function of regulatory T cells. In addition, selective targeting of individual Notch ligands and receptors can open a therapeutic window that does not exist with systemic pan-Notch inhibition. In some contexts, the discovery of new roles for understudied Notch pathway members, such as Notch3 and Notch4, may provide therapeutic opportunities even with prolonged inhibition, as the side effects of targeting these receptors are not predicted to be severe. Finally, combination therapies could provide other avenues, building on deeper molecular understanding of Notch signaling in immune cells.

## Author Contributions

FA and IM wrote and edited the manuscript. Both authors contributed to the article and approved the submitted version.

## Conflict of Interest

The authors declare that the research was conducted in the absence of any commercial or financial relationships that could be construed as a potential conflict of interest.

## References

[B1] Amsen D. Antov A. Jankovic D. Sher A. Radtke F. Souabni A. (2007). Direct regulation of Gata3 expression determines the T helper differentiation potential of Notch. *Immunity* 27 89–99. 10.1016/j.immuni.2007.05.021 17658279 PMC2062505

[B2] Amsen D. Blander J. M. Lee G. R. Tanigaki K. Honjo T. Flavell R. A. (2004). Instruction of distinct CD4 T helper cell fates by different notch ligands on antigen-presenting cells. *Cell* 117 515–526. 10.1016/s0092-8674(04)00451-9 15137944

[B3] Artavanis-Tsakonas S. Muskavitch M. A. Yedvobnick B. (1983). Molecular cloning of Notch, a locus affecting neurogenesis in *Drosophila melanogaster*. *Proc. Natl. Acad. Sci. U.S.A.* 80 1977–1981. 10.1073/pnas.80.7.1977 6403942 PMC393735

[B4] Aster J. C. Pear W. S. Blacklow S. C. (2017). The varied roles of notch in cancer. *Annu. Rev. Pathol.* 12 245–275. 10.1146/annurev-pathol-052016-100127 27959635 PMC5933931

[B5] Auderset F. Schuster S. Coutaz M. Koch U. Desgranges F. Merck E. (2012). Redundant Notch1 and Notch2 signaling is necessary for IFNgamma secretion by T helper 1 cells during infection with *Leishmania major*. *PLoS Pathog.* 8:e1002560. 10.1371/journal.ppat.1002560 22396647 PMC3291656

[B6] Backer R. A. Helbig C. Gentek R. Kent A. Laidlaw B. J. Dominguez C. X. (2014). A central role for Notch in effector CD8(+) T cell differentiation. *Nat. Immunol.* 15 1143–1151. 10.1038/ni.3027 25344724 PMC4232996

[B7] Bailis W. Yashiro-Ohtani Y. Fang T. C. Hatton R. D. Weaver C. T. Artis D. (2013). Notch simultaneously orchestrates multiple helper T cell programs independently of cytokine signals. *Immunity* 39 148–159. 10.1016/j.immuni.2013.07.006 23890069 PMC3762693

[B8] Bassil R. Zhu B. Lahoud Y. Riella L. V. Yagita H. Elyaman W. (2011). Notch ligand delta-like 4 blockade alleviates experimental autoimmune encephalomyelitis by promoting regulatory T cell development. *J. Immunol.* 187 2322–2328. 10.4049/jimmunol.1100725 21813770 PMC3311114

[B9] Best J. D. Smith D. W. Reilly M. A. O’Donnell R. Lewis H. D. Ellis S. (2007). The novel gamma secretase inhibitor N-[cis-4-[(4-chlorophenyl)sulfonyl]-4-(2,5-difluorophenyl)cyclohexyl]-1,1,1-trifluoromethanesulfonamide (MRK-560) reduces amyloid plaque deposition without evidence of notch-related pathology in the Tg2576 mouse. *J. Pharmacol. Exp. Ther.* 320 552–558. 10.1124/jpet.106.114330 17099072

[B10] Borgegard T. Gustavsson S. Nilsson C. Parpal S. Klintenberg R. Berg A. L. (2012). Alzheimer’s disease: presenilin 2-sparing gamma-secretase inhibition is a tolerable Abeta peptide-lowering strategy. *J. Neurosci.* 32 17297–17305. 10.1523/jneurosci.1451-12.2012 23197721 PMC6621836

[B11] Brandstadter J. D. Maillard I. (2019). Notch signalling in T cell homeostasis and differentiation. *Open Biol.* 9:190187. 10.1098/rsob.190187 31690218 PMC6893402

[B12] Briot A. Civelek M. Seki A. Hoi K. Mack J. J. Lee S. D. (2015). Endothelial NOTCH1 is suppressed by circulating lipids and antagonizes inflammation during atherosclerosis. *J. Exp. Med.* 212 2147–2163. 10.1084/jem.20150603 26552708 PMC4647265

[B13] Casulo C. Ruan J. Dang N. H. Gore L. Diefenbach C. Anne W. (2016). Safety and preliminary efficacy results of a phase i first-in-human study of the novel Notch-1 Targeting antibody brontictuzumab (OMP-52M51) administered intravenously to patients with hematologic malignancies. *Blood* 128:5108. 10.1182/blood.v128.22.5108.5108

[B14] Charbonnier L. M. Wang S. Georgiev P. Sefik E. Chatila T. A. (2015). Control of peripheral tolerance by regulatory T cell-intrinsic Notch signaling. *Nat. Immunol.* 16 1162–1173. 10.1038/ni.3288 26437242 PMC4618075

[B15] Chiorean E. G. LoRusso P. Strother R. M. Diamond J. R. Younger A. Messersmith W. A. (2015). A Phase I first-in-human study of enoticumab (REGN421), a fully human delta-like ligand 4 (Dll4) monoclonal antibody in patients with advanced solid tumors. *Clin. Cancer Res.* 21 2695–2703. 10.1158/1078-0432.ccr-14-2797 25724527

[B16] Choi W. H. Jo H. R. Jeon E. J. Youm S. Y. Jeon J. S. Son Y. G. (2016). Development, validation, and application of ELISA for detection of anti-HD105 antibodies in pre-clinical safety evaluation using monkeys. *J. Pharm. Biomed. Anal.* 131 309–315. 10.1016/j.jpba.2016.09.009 27619177

[B17] Choy L. Hagenbeek T. J. Solon M. French D. Finkle D. Shelton A. (2017). Constitutive NOTCH3 signaling promotes the growth of basal breast cancers. *Cancer Res.* 77 1439–1452. 10.1158/0008-5472.can-16-1022 28108512

[B18] Chung J. Ebens C. L. Perkey E. Radojcic V. Koch U. Scarpellino L. (2017). Fibroblastic niches prime T cell alloimmunity through Delta-like Notch ligands. *J. Clin. Invest.* 127 1574–1588. 10.1172/jci89535 28319044 PMC5373885

[B19] Chung J. Radojcic V. Perkey E. Parnell T. J. Niknafs Y. Jin X. (2019). Early notch signals induce a pathogenic molecular signature during priming of alloantigen-specific conventional CD4(+) T cells in graft-versus-host disease. *J. Immunol.* 203 557–568. 10.4049/jimmunol.1900192 31182480 PMC6615974

[B20] Churcher I. Beher D. Best J. D. Castro J. L. Clarke E. E. Gentry A. (2006). 4-substituted cyclohexyl sulfones as potent, orally active gamma-secretase inhibitors. *Bioorg. Med. Chem. Lett.* 16 280–284. 10.1016/j.bmcl.2005.10.009 16275079

[B21] Cook N. Basu B. Smith D. M. Gopinathan A. Evans J. Steward W. P. (2018). A phase I trial of the gamma-secretase inhibitor MK-0752 in combination with gemcitabine in patients with pancreatic ductal adenocarcinoma. *Br. J. Cancer* 118 793–801. 10.1038/bjc.2017.495 29438372 PMC5877439

[B22] Cullion K. Draheim K. M. Hermance N. Tammam J. Sharma V. M. Ware C. (2009). Targeting the Notch1 and mTOR pathways in a mouse T-ALL model. *Blood* 113 6172–6181. 10.1182/blood-2008-02-136762 19246562 PMC2699237

[B23] De Strooper B. Annaert W. Cupers P. Saftig P. Craessaerts K. Mumm J. S. (1999). A presenilin-1-dependent gamma-secretase-like protease mediates release of Notch intracellular domain. *Nature* 398 518–522. 10.1038/19083 10206645

[B24] Deangelo D. J. Stone R. M. Silverman L. B. Stock W. Attar E. C. Fearen I. (2006). A phase I clinical trial of the notch inhibitor MK-0752 in patients with T-cell acute lymphoblastic leukemia/lymphoma (T-ALL) and other leukemias. *J. Clin. Oncol.* 24 6585–6585. 10.1200/jco.2006.24.18_suppl.6585

[B25] del Amo F. F. Gendron-Maguire M. Swiatek P. J. Jenkins N. A. Copeland N. G. Gridley T. (1993). Cloning, analysis, and chromosomal localization of Notch-1, a mouse homolog of *Drosophila* Notch. *Genomics* 15 259–264. 10.1006/geno.1993.1055 8449489

[B26] Dexter J. S. (1914). The analysis of a case of continuous variation in *Drosophila* by a study of its linkage relations. *Am. Nat.* 48 712–758. 10.1086/279446

[B27] Dongre A. Surampudi L. Lawlor R. G. Fauq A. H. Miele L. Golde T. E. (2014). Non-canonical notch signaling drives activation and differentiation of peripheral CD4(+) T Cells. *Front. Immunol.* 5:54. 10.3389/fimmu.2014.00054 24611064 PMC3921607

[B28] Doody R. S. Raman R. Farlow M. Iwatsubo T. Vellas B. Joffe S. (2013). A phase 3 trial of semagacestat for treatment of Alzheimer’s disease. *N. Engl. J. Med.* 369 341–350. 10.1056/nejmoa1210951 23883379

[B29] Eixarch H. Mansilla M. J. Costa C. Kunkel S. L. Montalban X. Godessart N. (2013). Inhibition of delta-like ligand 4 decreases Th1/Th17 response in a mouse model of multiple sclerosis. *Neurosci. Lett.* 541 161–166. 10.1016/j.neulet.2013.02.038 23466692

[B30] El-Khoueiry A. B. Desai J. Padmanabhan Iyer S. Gadgeel S. M. Ramalingam S. S. Horn L. (2018). A phase I study of AL101, a pan-NOTCH inhibitor, in patients (pts) with locally advanced or metastatic solid tumors. *J. Clin. Oncol.* 36 2515–2515. 10.1200/jco.2018.36.15_suppl.2515

[B31] Ellisen L. W. Bird J. West D. C. Soreng A. L. Reynolds T. C. Smith S. D. (1991). TAN-1, the human homolog of the *Drosophila* notch gene, is broken by chromosomal translocations in T lymphoblastic neoplasms. *Cell* 66 649–661. 10.1016/0092-8674(91)90111-b 1831692

[B32] Elyaman W. Bassil R. Bradshaw E. M. Orent W. Lahoud Y. Zhu B. (2012). Notch receptors and Smad3 signaling cooperate in the induction of interleukin-9-producing T cells. *Immunity* 36 623–634. 10.1016/j.immuni.2012.01.020 22503540 PMC3572366

[B33] Fabbri G. Holmes A. B. Viganotti M. Scuoppo C. Belver L. Herranz D. (2017). Common nonmutational NOTCH1 activation in chronic lymphocytic leukemia. *Proc. Natl. Acad. Sci. U.S.A.* 114 E2911–E2919. 28314854 10.1073/pnas.1702564114 PMC5389283

[B34] Falchook G. S. Dowlati A. Naing A. Gribbin M. J. Jenkins D. W. Chang L. L. (2015). Phase I study of MEDI0639 in patients with advanced solid tumors. *J. Clin. Oncol.* 33 3024–3024. 10.1200/jco.2015.33.15_suppl.3024

[B35] Fang T. C. Yashiro-Ohtani Y. Del Bianco C. Knoblock D. M. Blacklow S. C. Pear W. S. (2007). Notch directly regulates Gata3 expression during T helper 2 cell differentiation. *Immunity* 27 100–110. 10.1016/j.immuni.2007.04.018 17658278 PMC2801546

[B36] Fasnacht N. Huang H. Y. Koch U. Favre S. Auderset F. Chai Q. (2014). Specific fibroblastic niches in secondary lymphoid organs orchestrate distinct Notch-regulated immune responses. *J. Exp. Med.* 211 2265–2279. 10.1084/jem.20132528 25311507 PMC4203954

[B37] Ferrarotto R. Eckhardt G. Patnaik A. LoRusso P. Faoro L. Heymach J. V. (2018). A phase I dose-escalation and dose-expansion study of brontictuzumab in subjects with selected solid tumors. *Ann. Oncol.* 29 1561–1568. 10.1093/annonc/mdy171 29726923

[B38] Ferrarotto R. Ho A. L. Wirth L. J. Dekel E. Walker R. W. Vergara-Silva A. L. (2019). ACCURACY: phase (P) 2 trial of AL101, a pan-Notch inhibitor, in patients (pts) with recurrent/metastatic (R/M) adenoid cystic carcinoma (ACC) with Notch activating mutations (Notchact mut). *J. Clin. Oncol.* 37:TS6098.

[B39] Folkman J. (1971). Tumor angiogenesis: therapeutic implications. *N. Engl. J. Med.* 285 1182–1186. 4938153 10.1056/NEJM197111182852108

[B40] Fu W. Lei C. Yu Y. Liu S. Li T. Lin F. (2019). EGFR/Notch antagonists enhance the response to inhibitors of the PI3K-Akt pathway by decreasing tumor-initiating cell frequency. *Clin. Cancer Res.* 25 2835–2847. 10.1158/1078-0432.ccr-18-2732 30670492

[B41] Fukuda D. Aikawa E. Swirski F. K. Novobrantseva T. I. Kotelianski V. Gorgun C. Z. (2012). Notch ligand delta-like 4 blockade attenuates atherosclerosis and metabolic disorders. *Proc. Natl. Acad. Sci. U.S.A.* 109 E1868–E1877. 22699504 10.1073/pnas.1116889109 PMC3390871

[B42] Gao W. Sweeney C. Connolly M. Kennedy A. Ng C. T. McCormick J. (2012). Notch-1 mediates hypoxia-induced angiogenesis in rheumatoid arthritis. *Arthritis Rheum* 64 2104–2113. 10.1002/art.34397 22275240

[B43] Haass C. Selkoe D. J. (1993). Cellular processing of beta-amyloid precursor protein and the genesis of amyloid beta-peptide. *Cell* 75 1039–1042. 10.1016/0092-8674(93)90312-e 8261505

[B44] Habets R. A. de Bock C. E. Serneels L. Lodewijckx I. Verbeke D. Nittner D. (2019). Safe targeting of T cell acute lymphoblastic leukemia by pathology-specific NOTCH inhibition. *Sci. Transl. Med.* 11:eaau6246. 10.1126/scitranslmed.aau6246 31142678

[B45] Harb H. Stephen-Victor E. Crestani E. Benamar M. Massoud A. Cui Y. (2020). A regulatory T cell Notch4–GDF15 axis licenses tissue inflammation in asthma. *Nat. Immunol.* 21 1359–1370. 10.1038/s41590-020-0777-3 32929274 PMC7578174

[B46] Hombrink P. Helbig C. Backer R. A. Piet B. Oja A. E. Stark R. (2016). Programs for the persistence, vigilance and control of human CD8(+) lung-resident memory T cells. *Nat. Immunol.* 17 1467–1478. 10.1038/ni.3589 27776108

[B47] Hozumi K. Mailhos C. Negishi N. Hirano K. Yahata T. Ando K. (2008). Delta-like 4 is indispensable in thymic environment specific for T cell development. *J. Exp. Med.* 205 2507–2513. 10.1084/jem.20080134 18824583 PMC2571926

[B48] Hu S. Fu W. Li T. Yuan Q. Wang F. Lv G. (2017). Antagonism of EGFR and Notch limits resistance to EGFR inhibitors and radiation by decreasing tumor-initiating cell frequency. *Sci. Transl. Med.* 9:eaag0339. 10.1126/scitranslmed.aag0339 28275151

[B49] Hu Z. I. Bendell J. C. Bullock A. LoConte N. K. Hatoum H. Ritch P. (2019). A randomized phase II trial of nab-paclitaxel and gemcitabine with tarextumab or placebo in patients with untreated metastatic pancreatic cancer. *Cancer Med.* 8 5148–5157. 10.1002/cam4.2425 31347292 PMC6718621

[B50] Huang M. T. Chen Y. L. Lien C. I. Liu W. L. Hsu L. C. Yagita H. (2017). Notch ligand DLL4 alleviates allergic airway inflammation via induction of a homeostatic regulatory pathway. *Sci. Rep.* 7:43535. 10.1038/srep43535 PMC5337933 28262821

[B51] Hurtado C. Safarova A. Smith M. Chung R. Bruyneel A. A. N. Gomez-Galeno J. (2019). Disruption of NOTCH signaling by a small molecule inhibitor of the transcription factor RBPJ. *Sci. Rep.* 9:10811. 10.1038/s41598-019-46948-5 PMC6658660 31346210

[B52] Jenkins D. W. Ross S. Veldman-Jones M. Foltz I. N. Clavette B. C. Manchulenko K. (2012). MEDI0639: a novel therapeutic antibody targeting Dll4 modulates endothelial cell function and angiogenesis in vivo. *Mol. Cancer Ther.* 11 1650–1660. 10.1158/1535-7163.mct-11-1027 22679110

[B53] Jia X. Wang W. Xu Z. Wang S. Wang T. Wang M. (2016). A humanized anti-DLL4 antibody promotes dysfunctional angiogenesis and inhibits breast tumor growth. *Sci. Rep.* 6:27985. 10.1038/srep27985 PMC4908374 27301650

[B54] Jimeno A. Moore K. N. Gordon M. Chugh R. Diamond J. R. Aljumaily R. (2019). A first-in-human phase 1a study of the bispecific anti-DLL4/anti-VEGF antibody navicixizumab (OMP-305B83) in patients with previously treated solid tumors. *Invest. New Drugs* 37 461–472. 10.1007/s10637-018-0665-y 30229512

[B55] Jurynczyk M. Jurewicz A. Bielecki B. Raine C. S. Selmaj K. (2005). Inhibition of Notch signaling enhances tissue repair in an animal model of multiple sclerosis. *J. Neuroimmunol.* 170 3–10. 10.1016/j.jneuroim.2005.10.013 16290267

[B56] Kang J. H. Kim B. S. Uhm T. G. Lee S. H. Lee G. R. Park C. S. (2009). Gamma-secretase inhibitor reduces allergic pulmonary inflammation by modulating Th1 and Th2 responses. *Am. J. Respir. Crit. Care Med.* 179 875–882. 10.1164/rccm.200806-893oc 19234107

[B57] Keck P. J. Hauser S. D. Krivi G. Sanzo K. Warren T. Feder J. (1989). Vascular permeability factor, an endothelial cell mitogen related to PDGF. *Science* 246 1309–1312. 10.1126/science.2479987 2479987

[B58] Kim D. H. Lee S. Kang H. G. Park H. W. Lee H. W. Kim D. (2020). Synergistic antitumor activity of a DLL4/VEGF bispecific therapeutic antibody in combination with irinotecan in gastric cancer. *BMB Rep.* 53 533–538. 10.5483/bmbrep.2020.53.10.103 32580836 PMC7607148

[B59] Kimberly W. T. LaVoie M. J. Ostaszewski B. L. Ye W. Wolfe M. S. Selkoe D. J. (2003). Gamma-secretase is a membrane protein complex comprised of presenilin, nicastrin. Aph-1, and Pen-2. *Proc. Natl. Acad. Sci. U.S.A.* 100 6382–6387. 10.1073/pnas.1037392100 12740439 PMC164455

[B60] KleinJan A. Tindemans I. Montgomery J. E. Lukkes M. de Bruijn M. J. W. van Nimwegen M. (2018). The Notch pathway inhibitor stapled alpha-helical peptide derived from mastermind-like 1 (SAHM1) abrogates the hallmarks of allergic asthma. *J. Allergy Clin. Immunol.* 142 76.e8–85.e8. 29111218 10.1016/j.jaci.2017.08.042 PMC7488091

[B61] Knoechel B. Roderick J. E. Williamson K. E. Zhu J. Lohr J. G. Cotton M. J. (2014). An epigenetic mechanism of resistance to targeted therapy in T cell acute lymphoblastic leukemia. *Nat. Genet.* 46 364–370. 24584072 10.1038/ng.2913 PMC4086945

[B62] Koch U. Fiorini E. Benedito R. Besseyrias V. Schuster-Gossler K. Pierres M. (2008). Delta-like 4 is the essential, nonredundant ligand for Notch1 during thymic T cell lineage commitment. *J. Exp. Med.* 205 2515–2523. 10.1084/jem.20080829 18824585 PMC2571927

[B63] Kopan R. Ilagan M. X. (2009). The canonical Notch signaling pathway: unfolding the activation mechanism. *Cell* 137 216–233. 10.1016/j.cell.2009.03.045 19379690 PMC2827930

[B64] Kovall R. A. Gebelein B. Sprinzak D. Kopan R. (2017). The canonical notch signaling pathway: structural and biochemical insights into shape. Sugar, and Force. *Dev. Cell* 41 228–241. 10.1016/j.devcel.2017.04.001 28486129 PMC5492985

[B65] Kummar S. Do K. T. O’Sullivan Coyne G. H. Turkbey B. Meltzer P. S. Polley E. (2015). Phase II trial of PF-03084014 in adults with desmoid tumors/aggressive fibromatosis. *J. Clin. Oncol.* 33:10563. 10.1200/jco.2015.33.15_suppl.10563 PMC5455706 28350521

[B66] Lafkas D. Shelton A. Chiu C. de Leon Boenig G. Chen Y. Stawicki S. S. (2015). Therapeutic antibodies reveal Notch control of transdifferentiation in the adult lung. *Nature* 528 127–131. 10.1038/nature15715 26580007

[B67] Laky K. Evans S. Perez-Diez A. Fowlkes B. J. (2015). Notch signaling regulates antigen sensitivity of naive CD4+ T cells by tuning co-stimulation. *Immunity* 42 80–94. 10.1016/j.immuni.2014.12.027 25607460 PMC4314725

[B68] Lardelli M. Dahlstrand J. Lendahl U. (1994). The novel Notch homologue mouse Notch 3 lacks specific epidermal growth factor-repeats and is expressed in proliferating neuroepithelium. *Mech. Dev.* 46 123–136. 10.1016/0925-4773(94)90081-7 7918097

[B69] Lee D. Kim D. Choi Y. B. Kang K. Sung E. S. Ahn J. H. (2016). Simultaneous blockade of VEGF and Dll4 by HD105, a bispecific antibody, inhibits tumor progression and angiogenesis. *MAbs* 8 892–904. 10.1080/19420862.2016.1171432 27049350 PMC4968104

[B70] Lee S. M. Moon J. Redman B. G. Chidiac T. Flaherty L. E. Zha Y. (2015). Phase 2 study of RO4929097, a gamma-secretase inhibitor, in metastatic melanoma: SWOG 0933. *Cancer* 121 432–440. 10.1002/cncr.29055 25250858 PMC4304973

[B71] Lehal R. Zaric J. Vigolo M. Urech C. Frismantas V. Zangger N. (2020). Pharmacological disruption of the Notch transcription factor complex. *Proc. Natl. Acad. Sci. U.S.A.* 117 16292–16301. 10.1073/pnas.1922606117 32601208 PMC7368267

[B72] Leung D. W. Cachianes G. Kuang W. J. Goeddel D. V. Ferrara N. (1989). Vascular endothelial growth factor is a secreted angiogenic mitogen. *Science* 246 1306–1309. 10.1126/science.2479986 2479986

[B73] Lewis K. L. Caton M. L. Bogunovic M. Greter M. Grajkowska L. T. Ng D. (2011). Notch2 receptor signaling controls functional differentiation of dendritic cells in the spleen and intestine. *Immunity* 35 780–791. 10.1016/j.immuni.2011.08.013 22018469 PMC3225703

[B74] Li Y. Hickson J. A. Ambrosi D. J. Haasch D. L. Foster-Duke K. D. Eaton L. J. (2018). ABT-165, a dual variable domain immunoglobulin (DVD-Ig) Targeting DLL4 and VEGF, demonstrates superior efficacy and favorable safety profiles in preclinical models. *Mol. Cancer Ther.* 17 1039–1050. 10.1158/1535-7163.mct-17-0800 29592882

[B75] Liang W. C. Wu X. Peale F. V. Lee C. V. Meng Y. G. Gutierrez J. (2006). Cross-species vascular endothelial growth factor (VEGF)-blocking antibodies completely inhibit the growth of human tumor xenografts and measure the contribution of stromal VEGF. *J. Biol. Chem.* 281 951–961. 10.1074/jbc.m508199200 16278208

[B76] Lopez-Lopez S. Monsalve E. M. Romero de Avila M. J. Gonzalez-Gomez J. Hernandez de Leon N. Ruiz-Marcos F. (2020). NOTCH3 signaling is essential for NF-kappaB activation in TLR-activated macrophages. *Sci. Rep.* 10:14839. 10.1038/s41598-020-71810-4 PMC7481794 32908186

[B77] Luistro L. He W. Smith M. Packman K. Vilenchik M. Carvajal D. (2009). Preclinical profile of a potent gamma-secretase inhibitor targeting notch signaling with in vivo efficacy and pharmacodynamic properties. *Cancer Res.* 69 7672–7680. 10.1158/0008-5472.can-09-1843 19773430 PMC5260798

[B78] Magee C. N. Murakami N. Borges T. J. Shimizu T. Safa K. Ohori S. (2019). Notch-1 inhibition promotes immune regulation in transplantation via regulatory T Cell-dependent mechanisms. *Circulation* 140 846–863. 10.1161/circulationaha.119.040563 31266349 PMC6722011

[B79] Maillard I. Weng A. P. Carpenter A. C. Rodriguez C. G. Sai H. Xu L. (2004). Mastermind critically regulates Notch-mediated lymphoid cell fate decisions. *Blood* 104 1696–1702. 10.1182/blood-2004-02-0514 15187027

[B80] Mancarella S. Serino G. Dituri F. Cigliano A. Ribback S. Wang J. (2020). Crenigacestat, a selective NOTCH1 inhibitor, reduces intrahepatic cholangiocarcinoma progression by blocking VEGFA/DLL4/MMP13 axis. *Cell Death Differ.* 27 2330–2343. 10.1038/s41418-020-0505-4 32042099 PMC7370218

[B81] Manderfield L. J. High F. A. Engleka K. A. Liu F. Li L. Rentschler S. (2012). Notch activation of Jagged1 contributes to the assembly of the arterial wall. *Circulation* 125 314–323. 10.1161/circulationaha.111.047159 22147907 PMC3260393

[B82] Massard C. Azaro A. Soria J. C. Lassen U. Le Tourneau C. Sarker D. (2018). First-in-human study of LY3039478, an oral Notch signaling inhibitor in advanced or metastatic cancer. *Ann. Oncol.* 29 1911–1917. 10.1093/annonc/mdy244 30060061

[B83] McKeage M. J. Kotasek D. Markman B. Hidalgo M. Millward M. J. Jameson M. B. (2018). Phase IB trial of the anti-cancer stem cell DLL4-binding agent demcizumab with pemetrexed and carboplatin as first-line treatment of metastatic non-squamous NSCLC. *Targeted Oncol.y* 13 89–98. 10.1007/s11523-017-0543-0 29188408

[B84] Messersmith W. A. Shapiro G. I. Cleary J. M. Jimeno A. Dasari A. Huang B. (2015). A Phase I, dose-finding study in patients with advanced solid malignancies of the oral gamma-secretase inhibitor PF-03084014. *Clin. Cancer Res.* 21 60–67. 10.1158/1078-0432.ccr-14-0607 25231399

[B85] Minter L. M. Turley D. M. Das P. Shin H. M. Joshi I. Lawlor R. G. (2005). Inhibitors of gamma-secretase block in vivo and in vitro T helper type 1 polarization by preventing Notch upregulation of Tbx21. *Nat. Immunol.* 6 680–688. 10.1038/ni1209x 15991363

[B86] Mochizuki K. Xie F. He S. Tong Q. Liu Y. Mochizuki I. (2013). Delta-like ligand 4 identifies a previously uncharacterized population of inflammatory dendritic cells that plays important roles in eliciting allogeneic T cell responses in mice. *J. Immunol.* 190 3772–3782. 10.4049/jimmunol.1202820 23440416 PMC3608722

[B87] Moellering R. E. Cornejo M. Davis T. N. Del Bianco C. Aster J. C. Blacklow S. C. (2009). Direct inhibition of the NOTCH transcription factor complex. *Nature* 462 182–188. 10.1038/nature08543 19907488 PMC2951323

[B88] Morgan T. H. (1917). The theory of the gene. *Am. Nat.* 51 513–544.

[B89] Morohashi Y. Kan T. Tominari Y. Fuwa H. Okamura Y. Watanabe N. (2006). C-terminal fragment of presenilin is the molecular target of a dipeptidic γ-secretase-specific inhibitor DAPT (N-[N-(3,5-Difluorophenacetyl)-L-alanyl]-S-phenylglycine t-Butyl Ester)^∗^. *J. Biol. Chem.* 281 14670–14676. 10.1074/jbc.m513012200 16569643

[B90] Nakazawa M. Ishii H. Aono H. Takai M. Honda T. Aratani S. (2001). Role of Notch-1 intracellular domain in activation of rheumatoid synoviocytes. *Arthritis Rheum* 44 1545–1554. 10.1002/1529-0131(200107)44:7<1545::aid-art278>3.0.co;2-q 11465706

[B91] Nam Y. Sliz P. Song L. Aster J. C. Blacklow S. C. (2006). Structural basis for cooperativity in recruitment of MAML coactivators to Notch transcription complexes. *Cell* 124 973–983. 10.1016/j.cell.2005.12.037 16530044

[B92] Noguera-Troise I. Daly C. Papadopoulos N. J. Coetzee S. Boland P. Gale N. W. (2006). Blockade of Dll4 inhibits tumour growth by promoting non-productive angiogenesis. *Nature* 444 1032–1037. 10.1038/nature05355 17183313

[B93] Okamoto M. Takeda K. Joetham A. Ohnishi H. Matsuda H. Swasey C. H. (2008). Essential role of Notch signaling in effector memory CD8+ T cell-mediated airway hyperresponsiveness and inflammation. *J. Exp. Med.* 205 1087–1097. 10.1084/jem.20072200 18426985 PMC2373841

[B94] Osborne B. A. Minter L. M. (2007). Notch signalling during peripheral T-cell activation and differentiation. *Nat. Rev. Immunol.* 7 64–75. 10.1038/nri1998 17170755

[B95] Pabois A. Devalliere J. Quillard T. Coulon F. Gerard N. Laboisse C. (2014). The disintegrin and metalloproteinase ADAM10 mediates a canonical Notch-dependent regulation of IL-6 through Dll4 in human endothelial cells. *Biochem. Pharmacol.* 91 510–521. 10.1016/j.bcp.2014.08.007 25130545

[B96] Pabois A. Pagie S. Gerard N. Laboisse C. Pattier S. Hulin P. (2016). Notch signaling mediates crosstalk between endothelial cells and macrophages via Dll4 and IL6 in cardiac microvascular inflammation. *Biochem. Pharmacol.* 104 95–107. 10.1016/j.bcp.2016.01.016 26826491

[B97] Palomero T. Sulis M. L. Cortina M. Real P. J. Barnes K. Ciofani M. (2007). Mutational loss of PTEN induces resistance to NOTCH1 inhibition in T-cell leukemia. *Nat. Med.* 13 1203–1210. 10.1038/nm1636 17873882 PMC2600418

[B98] Pant S. Jones S. F. Kurkjian C. D. Infante J. R. Moore K. N. Burris H. A. (2016). A first-in-human phase I study of the oral Notch inhibitor. LY900009, in patients with advanced cancer. *Eur. J. Cancer* 56 1–9. 10.1016/j.ejca.2015.11.021 26798966

[B99] Pardo-Saganta A. Tata P. R. Law B. M. Saez B. Chow R. D. Prabhu M. (2015). Parent stem cells can serve as niches for their daughter cells. *Nature* 523 597–601. 10.1038/nature14553 26147083 PMC4521991

[B100] Park J. S. Kim S. H. Kim K. Jin C. H. Choi K. Y. Jang J. (2015). Inhibition of notch signalling ameliorates experimental inflammatory arthritis. *Ann. Rheum Dis.* 74 267–274. 10.1136/annrheumdis-2013-203467 24255545

[B101] Perez-Fidalgo J. A. Ortega B. Simon S. Samartzis E. P. Boussios S. (2020). NOTCH signalling in ovarian cancer angiogenesis. *Ann. Transl. Med.* 8:1705. 10.21037/atm-20-4497 33490217 PMC7812236

[B102] Perkey E. Maurice De Sousa D. Carrington L. Chung J. Dils A. Granadier D. (2020). GCNT1-Mediated O-Glycosylation of the Sialomucin CD43 Is a Sensitive Indicator of Notch Signaling in Activated T Cells. *J. Immunol.* 204 1674–1688. 10.4049/jimmunol.1901194 32060138 PMC7306398

[B103] Petcherski A. G. Kimble J. (2000). LAG-3 is a putative transcriptional activator in the *C. elegans* Notch pathway. *Nature* 405 364–368. 10.1038/35012645 10830967

[B104] Pietanza M. C. Spira A. I. Jotte R. M. Gadgeel S. M. Mita A. C. Gluck L. L. H. W. L. (2015). Final results of phase Ib of tarextumab (TRXT, OMP-59R5, anti-Notch2/3) in combination with etoposide and platinum (EP) in patients (pts) with untreated extensive-stage small-cell lung cancer (ED-SCLC). *J. Clin. Oncol.* 33 7508–7508. 10.1200/jco.2015.33.15_suppl.7508

[B105] Pui J. C. Allman D. Xu L. DeRocco S. Karnell F. G. Bakkour S. (1999). Notch1 expression in early lymphopoiesis influences B versus T lineage determination. *Immunity* 11 299–308. 10.1016/s1074-7613(00)80105-3 10514008

[B106] Radojcic V. Paz K. Chung J. Du J. Perkey E. T. Flynn R. (2018). Notch signaling mediated by Delta-like ligands 1 and 4 controls the pathogenesis of chronic GVHD in mice. *Blood* 132 2188–2200. 10.1182/blood-2018-03-841155 30181175 PMC6238189

[B107] Radtke F. Fasnacht N. Macdonald H. R. (2010). Notch signaling in the immune system. *Immunity* 32 14–27. 10.1016/j.immuni.2010.01.004 20152168

[B108] Radtke F. Wilson A. Stark G. Bauer M. van Meerwijk J. MacDonald H. R. (1999). Deficient T cell fate specification in mice with an induced inactivation of Notch1. *Immunity* 10 547–558. 10.1016/s1074-7613(00)80054-0 10367900

[B109] Ramakrishnan V. Ansell S. Haug J. Grote D. Kimlinger T. Stenson M. (2012). MRK003, a γ-secretase inhibitor exhibits promising in vitro pre-clinical activity in multiple myeloma and non-Hodgkin’s lymphoma. *Leukemia* 26 340–348. 10.1038/leu.2011.192 21826062 PMC4384189

[B110] Real P. J. Tosello V. Palomero T. Castillo M. Hernando E. de Stanchina E. (2009). Gamma-secretase inhibitors reverse glucocorticoid resistance in T cell acute lymphoblastic leukemia. *Nat. Med.* 15 50–58. 10.1038/nm.1900 19098907 PMC2692090

[B111] Reynolds N. D. Lukacs N. W. Long N. Karpus W. J. (2011). Delta-like ligand 4 regulates central nervous system T cell accumulation during experimental autoimmune encephalomyelitis. *J. Immunol.* 187 2803–2813. 10.4049/jimmunol.1100160 21788444 PMC3159801

[B112] Riccio O. van Gijn M. E. Bezdek A. C. Pellegrinet L. van Es J. H. Zimber-Strobl U. (2008). Loss of intestinal crypt progenitor cells owing to inactivation of both Notch1 and Notch2 is accompanied by derepression of CDK inhibitors p27Kip1 and p57Kip2. *EMBO Rep.* 9 377–383. 10.1038/embor.2008.7 18274550 PMC2288761

[B113] Ridgway J. Zhang G. Wu Y. Stawicki S. Liang W. C. Chanthery Y. (2006). Inhibition of Dll4 signalling inhibits tumour growth by deregulating angiogenesis. *Nature* 444 1083–1087. 10.1038/nature05313 17183323

[B114] Riella L. V. Ueno T. Batal I. De Serres S. A. Bassil R. Elyaman W. (2011). Blockade of Notch ligand delta1 promotes allograft survival by inhibiting alloreactive Th1 cells and cytotoxic T cell generation. *J. Immunol.* 187 4629–4638. 10.4049/jimmunol.1004076 21949024 PMC3197868

[B115] Riella L. V. Yang J. Chock S. Safa K. Magee C. N. Vanguri V. (2013). Jagged2-signaling promotes IL-6-dependent transplant rejection. *Eur. J. Immunol.* 43 1449–1458. 10.1002/eji.201243151 23526606

[B116] Roderick J. E. Gonzalez-Perez G. Kuksin C. A. Dongre A. Roberts E. R. Srinivasan J. (2013). Therapeutic targeting of NOTCH signaling ameliorates immune-mediated bone marrow failure of aplastic anemia. *J. Exp. Med.* 210 1311–1329. 10.1084/jem.20112615 23733784 PMC3698520

[B117] Sahebjam S. Bedard P. L. Castonguay V. Chen Z. Reedijk M. Liu G. (2013). A phase I study of the combination of ro4929097 and cediranib in patients with advanced solid tumours (PJC-004/NCI 8503). *Br. J. Cancer* 109 943–949. 10.1038/bjc.2013.380 23868004 PMC3749563

[B118] Saito T. Chiba S. Ichikawa M. Kunisato A. Asai T. Shimizu K. (2003). Notch2 is preferentially expressed in mature B cells and indispensable for marginal zone B lineage development. *Immunity* 18 675–685. 10.1016/s1074-7613(03)00111-0 12753744

[B119] Samon J. B. Castillo-Martin M. Hadler M. Ambesi-Impiobato A. Paietta E. Racevskis J. (2012). Preclinical analysis of the gamma-secretase inhibitor PF-03084014 in combination with glucocorticoids in T-cell acute lymphoblastic leukemia. *Mol. Cancer Ther.* 11 1565–1575. 10.1158/1535-7163.mct-11-0938 22504949 PMC3392513

[B120] Sanchez-Martin M. Ambesi-Impiombato A. Qin Y. Herranz D. Bansal M. Girardi T. (2017). Synergistic antileukemic therapies in NOTCH1-induced T-ALL. *Proc. Natl. Acad. Sci. U.S.A.* 114 2006–2011. 10.1073/pnas.1611831114 28174276 PMC5338362

[B121] Sandy A. R. Chung J. Toubai T. Shan G. T. Tran I. T. Friedman A., et al. (2013a). T cell-specific notch inhibition blocks graft-versus-host disease by inducing a hyporesponsive program in alloreactive CD4+ and CD8+ T cells. *J. Immunol.* 190 5818–5828. 10.4049/jimmunol.1203452 23636056 PMC3660433

[B122] Sandy A. R. Stoolman J. Malott K. Pongtornpipat P. Segal B. M. Maillard I. (2013b). Notch signaling regulates T cell accumulation and function in the central nervous system during experimental autoimmune encephalomyelitis. *J. Immunol.* 191 1606–1613. 10.4049/jimmunol.1301116 23825310 PMC3735619

[B123] Sato T. van Es J. H. Snippert H. J. Stange D. E. Vries R. G. van den Born M. (2011). Paneth cells constitute the niche for Lgr5 stem cells in intestinal crypts. *Nature* 469 415–418. 10.1038/nature09637 21113151 PMC3547360

[B124] Satpathy A. T. Briseno C. G. Lee J. S. Ng D. Manieri N. A. Kc W. (2013). Notch2-dependent classical dendritic cells orchestrate intestinal immunity to attaching-and-effacing bacterial pathogens. *Nat. Immunol.* 14 937–948. 10.1038/ni.2679 23913046 PMC3788683

[B125] Schaller M. A. Neupane R. Rudd B. D. Kunkel S. L. Kallal L. E. Lincoln P. (2007). Notch ligand Delta-like 4 regulates disease pathogenesis during respiratory viral infections by modulating Th2 cytokines. *J. Exp. Med.* 204 2925–2934. 10.1084/jem.20070661 17998388 PMC2118527

[B126] Shin H. M. Minter L. M. Cho O. H. Gottipati S. Fauq A. H. Golde T. E. (2006). Notch1 augments NF-kappaB activity by facilitating its nuclear retention. *EMBO J.* 25 129–138. 10.1038/sj.emboj.7600902 16319921 PMC1356346

[B127] Shin H. M. Tilahun M. E. Cho O. H. Chandiran K. Kuksin C. A. Keerthivasan S. (2014). NOTCH1 Can Initiate NF-kappaB activation via cytosolic interactions with components of the T Cell Signalosome. *Front. Immunol.* 5:249. 10.3389/fimmu.2014.00249 24904593 PMC4033603

[B128] Smith D. C. Eisenberg P. D. Manikhas G. Chugh R. Gubens M. A. Stagg R. J. (2014). A phase I dose escalation and expansion study of the anticancer stem cell agent demcizumab (anti-DLL4) in patients with previously treated solid tumors. *Clin. Cancer Res.* 20 6295–6303. 10.1158/1078-0432.ccr-14-1373 25324140

[B129] Sosa Iglesias V. Giuranno L. Dubois L. J. Theys J. Vooijs M. (2018). Drug resistance in non-small cell lung cancer: a potential for NOTCH targeting? *Front. Oncol.* 8:267. 10.3389/fonc.2018.00267 30087852 PMC6066509

[B130] Sparling D. P. McCullough N. Pajvani U. Humphrey M. B. (2020). Inhibition of γ-secretase in adipocytes leads to altered IL-6 secretion and adipose inflammation. *Adipocyte* 9 326–335. 10.1080/21623945.2020.1788235 32603641 PMC7469479

[B131] Takam Kamga P. Dal Collo G. Midolo M. Adamo A. Delfino P. Mercuri A. (2019). Inhibition of notch signaling enhances chemosensitivity in b-cell precursor acute lymphoblastic leukemia. *Cancer Res.* 79 639–649. 10.1158/0008-5472.can-18-1617 30563887

[B132] Tamura K. Taniguchi Y. Minoguchi S. Sakai T. Tun T. Furukawa T. (1995). Physical interaction between a novel domain of the receptor Notch and the transcription factor RBP-J kappa/Su(H). *Curr. Biol.* 5 1416–1423. 10.1016/s0960-9822(95)00279-x 8749394

[B133] Tanaka S. Nakada M. Yamada D. Nakano I. Todo T. Ino Y. (2015). Strong therapeutic potential of γ-secretase inhibitor MRK003 for CD44-high and CD133-low glioblastoma initiating cells. *J. Neuro Oncol.* 121 239–250. 10.1007/s11060-014-1630-z 25293440

[B134] Tanaka S. Tsukada J. Suzuki W. Hayashi K. Tanigaki K. Tsuji M. (2006). The interleukin-4 enhancer CNS-2 is regulated by Notch signals and controls initial expression in NKT cells and memory-type CD4 T cells. *Immunity* 24 689–701. 10.1016/j.immuni.2006.04.009 16782026

[B135] Tanigaki K. Han H. Yamamoto N. Tashiro K. Ikegawa M. Kuroda K. (2002). Notch-RBP-J signaling is involved in cell fate determination of marginal zone B cells. *Nat. Immunol.* 3 443–450. 10.1038/ni793 11967543

[B136] Tatarek J. Cullion K. Ashworth T. Gerstein R. Aster J. C. Kelliher M. A. (2011). Notch1 inhibition targets the leukemia-initiating cells in a Tal1/Lmo2 mouse model of T-ALL. *Blood* 118 1579–1590. 10.1182/blood-2010-08-300343 21670468 PMC3156046

[B137] Tindemans I. van Schoonhoven A. KleinJan A. de Bruijn M. J. Lukkes M. van Nimwegen M. (2020). Notch signaling licenses allergic airway inflammation by promoting Th2 cell lymph node egress. *J. Clin. Invest.* 130 3576–3591. 10.1172/jci128310 32255764 PMC7324208

[B138] Tkachev V. Kuhnert F. Furlan S. N. Zheng H. Hunt D. J. Colonna L. (2018). Pharmacologic blockade of Notch/Delta-like ligand 4 signaling protects from gastrointestinal acute graft-versus-host disease in non-human primates. *Blood* 132:2027. 10.1182/blood-2018-99-110030

[B139] Tran I. T. Sandy A. R. Carulli A. J. Ebens C. Chung J. Shan G. T. (2013). Blockade of individual Notch ligands and receptors controls graft-versus-host disease. *J. Clin. Invest.* 123 1590–1604. 10.1172/jci65477 23454750 PMC3613915

[B140] Tu L. Fang T. C. Artis D. Shestova O. Pross S. E. Maillard I. (2005). Notch signaling is an important regulator of type 2 immunity. *J. Exp. Med*, 202 1037–1042. 10.1084/jem.20050923 16230473 PMC2213210

[B141] Uyttendaele H. Marazzi G. Wu G. Yan Q. Sassoon D. Kitajewski J. (1996). Notch4/int-3, a mammary proto-oncogene, is an endothelial cell-specific mammalian Notch gene. *Development* 122 2251–2259. 10.1242/dev.122.7.2251 8681805

[B142] van Es J. H. van Gijn M. E. Riccio O. van den Born M. Vooijs M. Begthel H. (2005). Notch/gamma-secretase inhibition turns proliferative cells in intestinal crypts and adenomas into goblet cells. *Nature* 435 959–963. 10.1038/nature03659 15959515

[B143] VanDussen K. L. Carulli A. J. Keeley T. M. Patel S. R. Puthoff B. J. Magness S. T. (2012). Notch signaling modulates proliferation and differentiation of intestinal crypt base columnar stem cells. *Development* 139 488–497. 10.1242/dev.070763 22190634 PMC3252352

[B144] Vigouroux S. Yvon E. Wagner H. J. Biagi E. Dotti G. Sili U. (2003). Induction of antigen-specific regulatory T cells following overexpression of a Notch ligand by human B lymphocytes. *J. Virol.* 77 10872–10880. 10.1128/jvi.77.20.10872-10880.2003 14512537 PMC224961

[B145] Wainberg Z. Strickler J. Gordon M. Barve M. Wang L. Yue H. (2018). P-234 - Phase 1b open-label study evaluating the safety, pharmacokinetics, and preliminary efficacy of ABT-165 plus FOLFIRI in patients with second-line (2L) colorectal cancer (CRC). *Ann. Oncol.* 29:v66. 10.1093/annonc/mdy151.233

[B146] Wei K. Korsunsky I. Marshall J. L. Gao A. Watts G. F. M. Major T. (2020). Notch signalling drives synovial fibroblast identity and arthritis pathology. *Nature* 582 259–264. 10.1038/s41586-020-2222-z 32499639 PMC7841716

[B147] Weinmaster G. Roberts V. J. Lemke G. (1992). Notch2: a second mammalian Notch gene. *Development* 116 931–941. 10.1242/dev.116.4.931 1295745

[B148] Weng A. P. Ferrando A. A. Lee W. Morris J. P. IV Silverman L. B. Sanchez-Irizarry C. (2004). Activating mutations of NOTCH1 in human T cell acute lymphoblastic leukemia. *Science* 306 269–271. 10.1126/science.1102160 15472075

[B149] Weng A. P. Millholland J. M. Yashiro-Ohtani Y. Arcangeli M. L. Lau A. Wai C. (2006). c-Myc is an important direct target of Notch1 in T-cell acute lymphoblastic leukemia/lymphoma. *Genes Dev.* 20 2096–2109. 10.1101/gad.1450406 16847353 PMC1536060

[B150] Whitehead J. Thygesen H. Jaki T. Davies S. Halford S. Turner H. (2012). A novel Phase I/IIa design for early phase oncology studies and its application in the evaluation of MK-0752 in pancreatic cancer. *Stat. Med.* 31 1931–1943. 10.1002/sim.5331 22495759

[B151] Wilson J. J. Kovall R. A. (2006). Crystal structure of the CSL-Notch-mastermind ternary complex bound to DNA. *Cell* 124 985–996. 10.1016/j.cell.2006.01.035 16530045

[B152] Wolfe M. S. (2020). Unraveling the complexity of gamma-secretase. *Semin. Cell Dev. Biol.* 105 3–11. 10.1016/j.semcdb.2020.01.005 31980377 PMC7371508

[B153] Wong K. K. Carpenter M. J. Young L. L. Walker S. J. McKenzie G. Rust A. J. (2003). Notch ligation by Delta1 inhibits peripheral immune responses to transplantation antigens by a CD8+ cell-dependent mechanism. *J. Clin. Invest.* 112 1741–1750. 10.1172/jci200318020 14660750 PMC281641

[B154] Wood S. Feng J. Chung J. Radojcic V. Sandy-Sloat A. R. Friedman A. (2015). Transient blockade of delta-like Notch ligands prevents allograft rejection mediated by cellular and humoral mechanisms in a mouse model of heart transplantation. *J. Immunol.* 194 2899–2908. 10.4049/jimmunol.1402034 25687759 PMC4355388

[B155] Wu L. Aster J. C. Blacklow S. C. Lake R. Artavanis-Tsakonas S. Griffin J. D. (2000). MAML1, a human homologue of *Drosophila* mastermind, is a transcriptional co-activator for NOTCH receptors. *Nat. Genet.* 26 484–489. 10.1038/82644 11101851

[B156] Wu Y. Cain-Hom C. Choy L. Hagenbeek T. J. de Leon G. P. Chen Y. (2010). Therapeutic antibody targeting of individual Notch receptors. *Nature* 464 1052–1057. 10.1038/nature08878 20393564

[B157] Xia M. Viera-Hutchins L. Garcia-Lloret M. Noval Rivas M. Wise P. McGhee S. A. (2015). Vehicular exhaust particles promote allergic airway inflammation through an aryl hydrocarbon receptor-notch signaling cascade. *J. Allergy Clin. Immunol.* 136 441–453. 10.1016/j.jaci.2015.02.014 25825216 PMC4530027

[B158] Xu Z. Wang Z. Jia X. Wang L. Chen Z. Wang S. (2016). MMGZ01, an anti-DLL4 monoclonal antibody, promotes nonfunctional vessels and inhibits breast tumor growth. *Cancer Lett.* 372 118–127. 10.1016/j.canlet.2015.12.025 26739060

[B159] Yamanda S. Ebihara S. Asada M. Okazaki T. Niu K. Ebihara T. (2009). Role of ephrinB2 in nonproductive angiogenesis induced by Delta-like 4 blockade. *Blood* 113 3631–3639. 10.1182/blood-2008-07-170381 19218547

[B160] Yan M. Callahan C. A. Beyer J. C. Allamneni K. P. Zhang G. Ridgway J. B. (2010). Chronic DLL4 blockade induces vascular neoplasms. *Nature* 463 E6–E7. 20147986 10.1038/nature08751

[B161] Yang Q. Monticelli L. A. Saenz S. A. Chi A. W. Sonnenberg G. F. Tang J. (2013). T cell factor 1 is required for group 2 innate lymphoid cell generation. *Immunity* 38 694–704. 10.1016/j.immuni.2012.12.003 23601684 PMC4029843

[B162] Yang Z. J. Yu Z. Y. Cai Y. M. Du R. R. Cai L. (2020). Engineering of an enhanced synthetic Notch receptor by reducing ligand-independent activation. *Commun Biol.* 3:116. 10.1038/s42003-020-0848-x PMC7069970 32170210

[B163] Yen W. C. Fischer M. M. Axelrod F. Bond C. Cain J. Cancilla B. (2015). Targeting Notch signaling with a Notch2/Notch3 antagonist (tarextumab) inhibits tumor growth and decreases tumor-initiating cell frequency. *Clin. Cancer Res.* 21 2084–2095. 10.1158/1078-0432.ccr-14-2808 25934888

[B164] Yeom D. H. Lee Y. S. Ryu I. Lee S. Sung B. Lee H. B. (2021). ABL001, a Bispecific antibody targeting VEGF and DLL4, with chemotherapy, synergistically inhibits tumor progression in xenograft models. *Int. J. Mol. Sci.* 22:241. 10.3390/ijms22010241 33383646 PMC7796106

[B165] Yuan J. S. Kousis P. C. Suliman S. Visan I. Guidos C. J. (2010). Functions of notch signaling in the immune system: consensus and controversies. *Annu. Rev. Immunol.* 28 343–365. 10.1146/annurev.immunol.021908.132719 20192807

[B166] Yvon E. S. Vigouroux S. Rousseau R. F. Biagi E. Amrolia P. Dotti G. (2003). Overexpression of the Notch ligand. Jagged-1, induces alloantigen-specific human regulatory T cells. *Blood* 102 3815–3821. 10.1182/blood-2002-12-3826 12842995

[B167] Zhang K. Xu X. Pasha M. A. Siebel C. W. Costello A. Haczku A. (2017). Cutting edge: notch signaling promotes the plasticity of Group-2 innate lymphoid cells. *J. Immunol.* 198 1798–1803. 10.4049/jimmunol.1601421 28115527 PMC5321819

[B168] Zhang W. Zhang X. Sheng A. Weng C. Zhu T. Zhao W. (2015). gamma-secretase inhibitor alleviates acute airway inflammation of allergic asthma in mice by downregulating Th17 cell differentiation. *Mediators Inflamm* 2015:258168. 10.1155/2015/258168 PMC4539120 26339131

[B169] Zhang Y. Sandy A. R. Wang J. Radojcic V. Shan G. T. I Tran T. (2011). Notch signaling is a critical regulator of allogeneic CD4+ T-cell responses mediating graft-versus-host disease. *Blood* 117 299–308. 10.1182/blood-2010-03-271940 20870902 PMC3037751

[B170] Zheng H. Bae Y. Kasimir-Bauer S. Tang R. Chen J. Ren G. (2017). Therapeutic antibody targeting tumor- and osteoblastic niche-derived jagged1 sensitizes bone metastasis to chemotherapy. *Cancer Cell* 32 731.e6–747.e6. 29232552 10.1016/j.ccell.2017.11.002 PMC5729937

